# Analyses of *Catharanthus roseus* and *Arabidopsis thaliana* WRKY transcription factors reveal involvement in jasmonate signaling

**DOI:** 10.1186/1471-2164-15-502

**Published:** 2014-06-20

**Authors:** Craig Schluttenhofer, Sitakanta Pattanaik, Barunava Patra, Ling Yuan

**Affiliations:** Department of Plant and Soil Science, University of Kentucky, Lexington, KY 40546 USA; Kentucky Tobacco Research and Development Center, University of Kentucky, Lexington, KY 40546 USA

**Keywords:** *Catharanthus roseus*, Terpenoid indole alkaloid, Transcriptome, Secondary metabolism, WRKY transcription factors

## Abstract

**Background:**

To combat infection to biotic stress plants elicit the biosynthesis of numerous natural products, many of which are valuable pharmaceutical compounds. Jasmonate is a central regulator of defense response to pathogens and accumulation of specialized metabolites. *Catharanthus roseus* produces a large number of terpenoid indole alkaloids (TIAs) and is an excellent model for understanding the regulation of this class of valuable compounds. Recent work illustrates a possible role for the *Catharanthus* WRKY transcription factors (TFs) in regulating TIA biosynthesis. In Arabidopsis and other plants, the WRKY TF family is also shown to play important role in controlling tolerance to biotic and abiotic stresses, as well as secondary metabolism.

**Results:**

Here, we describe the WRKY TF families in response to jasmonate in Arabidopsis and *Catharanthus*. Publically available Arabidopsis microarrays revealed at least 30% (22 of 72) of WRKY TFs respond to jasmonate treatments. Microarray analysis identified at least six jasmonate responsive Arabidopsis WRKY genes (*AtWRKY7*, *AtWRKY20*, *AtWRKY26*, *AtWRKY45*, *AtWRKY48*, and *AtWRKY72*) that have not been previously reported. The *Catharanthus* WRKY TF family is comprised of at least 48 members. Phylogenetic clustering reveals 11 group I, 32 group II, and 5 group III WRKY TFs. Furthermore, we found that at least 25% (12 of 48) were jasmonate responsive, and 75% (9 of 12) of the jasmonate responsive CrWRKYs are orthologs of AtWRKYs known to be regulated by jasmonate.

**Conclusion:**

Overall, the CrWRKY family, ascertained from transcriptome sequences, contains approximately 75% of the number of WRKYs found in other sequenced asterid species (pepper, tomato, potato, and bladderwort). Microarray and transcriptomic data indicate that expression of WRKY TFs in Arabidopsis and *Catharanthus* are under tight spatio-temporal and developmental control, and potentially have a significant role in jasmonate signaling. Profiling of CrWRKY expression in response to jasmonate treatment revealed potential associations with secondary metabolism. This study provides a foundation for further characterization of WRKY TFs in jasmonate responses and regulation of natural product biosynthesis.

**Electronic supplementary material:**

The online version of this article (doi:10.1186/1471-2164-15-502) contains supplementary material, which is available to authorized users.

## Background

Secondary metabolites (a.k.a natural products or specialized metabolites), are compounds synthesized by plants for attracting pollinators [1], inter-plant communication [2], and defense [3]. The plethora of natural products synthesized by plants also provides many valuable pharmaceutical compounds. *Catharanthus roseus* (L.) G. Don, commonly called Madagascar periwinkle or annual vinca, belongs to the Apocynaceae family and synthesizes over 130 terpene indole alkaloids (TIAs) including the pharmaceutically valuable vinblastine and vincristine. Vinblastine and vincristine provide antineoplastic compounds effective in the treatment of several types of cancer [4]. The biosynthesis of these compounds, along with other TIAs, is regulated by UV light [5, 6], fungal elicitors [7], wounding [8, 9], drought [10], cold [11, 12] and salt stress [12]. A principal elicitor of TIA production in *Catharanthus*, as well as natural products in many other medicinal species, is the phytohormone jasmonate which functions in plant defense signaling to protect the plant from biotic stresses [3, 13].

Transcription factors (TFs) play a critical role in responding to jasmonate to elicit the synthesis of TIAs in *Catharanthus* [14]– [16]. Negative regulators also mediate jasmonate signaling of the TIA pathway in *Catharanthus* [17, 18]. In plants, WRKY TFs are critical regulators of response to biotic and abiotic stress. WRKY TFs have been attributed to tolerance of drought [19], salt [20], nutrient deficiency [21], osmotic [22], cold [23], heat [24], oxidative [25], wounding [26], pathogens [27], and UV-B stresses [28]. The WRKY TF family is primarily a plant specific family with the exception of several examples in protozoa [29]. The WRKY domain is a 60 to 70 amino acid long DNA binding domain that recognizes the W-box (TTGACC/T); however, recent studies suggest this *cis*-element may be more degenerate and other components are involved for WRKY binding to DNA in response to a specific stimulus [30, 31]. The N-terminal portion of the WRKY domain is characterized by a highly conserved WRKYGQK motif whereas the C-terminal region of the domain contains either a Cys_2_-His_2_ or Cys_2_-His-Cys zinc-finger [32]. WRKY TFs are distinguished by the presence of one or two WRKY domains. Group I WRKYs typically contain two WRKY domains whereas group II and group III members only contain one WRKY domain [32]. Up to five subgroups (IIa, IIb, IIc, IId, and IIe) are recognized in the group II WRKY TFs [32].

In Arabidopsis, WRKY TFs are well established in salicylic acid (SA) and defense signaling pathways [33]– [35]. The majority of Arabidopsis WRKY TFs are induced by treatment with SA [33]. However, the importance of WRKY TFs in JA signaling network is relatively less studied. Li et al. [36] identified AtWRKY70 as a positive regulator of SA signaling and negative regulator of jasmonate signaling. Mutation of *AtWRKY33* enhances susceptibility to necrotrophic pathogens by up-regulating JAZ proteins, repressors of jasmonate signaling [27, 37]. Jasmonate positively regulates *AtWRKY18* and *AtWRKY40* which also negatively regulate abscisic acid (ABA) response [38]. *AtWRKY6*, *AtWRKY8*, *AtWRKY11*, *AtWRKY17*, *AtWRKY25*, *AtWRKY28*, *AtWRKY38*, *AtWRKY60*, *AtWRKY62*, and *AtWRKY70* are also differentially expressed by jasmonates to regulate plant defense [22, 39]– [45].

Over the last several years, WRKY TFs have emerged as a key family in the induction of natural product biosynthesis [46, 47]. CjWRKY1, from *Coptis japonica*, regulates the production of the benzylisoquinoline alkaloid, berberine [48]. Cotton (*Gossypium arboreum*) GaWRKY1 affects the biosynthesis of the sesquiterpene, gossypol [49]. Multiple biosynthetic genes for the sesquiterpene lactone, artemisinin, valuable as an anti-malaria drug, are regulated by *Artemisia annua* WRKY1 [50]. *Hevea brasiliensis* WRKY1 is present in the latex of mechanically wounded (tapped) trees suggesting involvement in rubber latex synthesis [51]. The *Taxus chinensis* WRKY1 was found to regulate the expression of *10-DEACETYLBACCATIN III-10 B-O-ACETYL TRANSFERASE* (DBAT), a gene encoding a key enzyme catalyzing a rate limiting step in the biosynthesis of the anticancer terpene, paclitaxel [47]. In Arabidopsis, camalexin biosynthesis is mis-regulated in *wrky33* mutant [52]. Over-expression in Arabidopsis of *Panax quinquefolius* WRKY1, a jasmonate responsive WRKY from American ginseng, is found to enhance expression of genes related to drought, salt, and disease resistance, leading to improvement of seedling survival to drought and salt stress, in addition to regulating the expression of genes related to triterpene biosynthesis [53]. In *Catharanthus*, *CrWRKY1* has been demonstrated to respond to jasmonate, ethylene, and gibberellin signaling to regulate TIA production [46]. Over-expression of *CrWRKY1* increased the production of serpentine while simultaneously decreasing catharanthine accumulation, suggesting this WRKY may function in governing gene expression that specifically directs the flow of metabolites to synthesize TIAs in *Catharanthus* roots.

Identification of jasmonate responsive WRKY will thus provide useful information on plant defense and natural product regulatory networks. Understanding the number and types of WRKY TFs present in *Catharanthus* will provide a clearer picture on the regulation of TIAs by this important TF family. Here, we present jasmonate responsive WRKYs from Arabidopsis and *Catharanthus*. First, we analyzed Arabidopsis microarray data to help identify the involvement of the WRKY TFs in jasmonate signaling. We then used the medicinal plant transcriptome data to identify the *Catharanthus* family of WRKY TFs. Expression data from *Catharanthus* revealed the induction of multiple WRKY transcripts by methyl jasmonate (MeJA) treatment. Seventy-five percent of the jasmonate responsive CrWRKYs are orthologs of AtWRKYs known to be regulated by jasmonate. MeJA-induced WRKYs provide potential candidates for further regulation of TIA accumulation in *Catharanthus*. The identification of orthologs for WRKY TFs known to be involved in specialized metabolism in other plant species indicates the possible involvement of additional WRKY TFs in regulation of TIA production in *Catharanthus*.

## Results and discussion

### WRKY TFs are involved in Jasmonate signaling

The role of WRKY TF family in SA signaling and plant defense is well established and has been systematically analyzed in Arabidopsis, but remains less clear for jasmonate signaling. Jasmonate is a key phytohormone regulating the production of specialized metabolites in many plant species, including *Catharanthus*. While Arabidopsis does not synthesize TIAs as found in *Catharanthus*, studying AtWRKYs can answer several important questions. First we wanted to determine whether the WRKY family is important for regulating jasmonate signaling in a model species, such as Arabidopsis. Second, we wanted to elucidate jasmonate responsive AtWRKYs to aid the identification of CrWRKY orthologs with potentially conserved regulatory functions. Comparison of orthologous jasmonate responsive WRKYs from Arabidopsis and *Catharanthus* will identify WRKYs that are potentially involved in modulating jasmonate signaling, and in turn identify candidates that regulate TIA production.

To clearly establish the role of WRKY TFs in jasmonate signaling we first identified jasmonate responsive WRKYs in the model plant Arabidopsis. To ascertain jasmonate responsive Arabidopsis WRKY TFs we used publically available microarray datasets (Table 1). The ATH1 Affymetrix arrays used contain probes identifying 85% (61 of the 72) of Arabidopsis WRKY TFs (Additional file 1: Table S1). From five datasets, we identified 39 AtWRKY TFs that significantly change in response to jasmonate treatment (Additional file 2: Table S2). Of the 39 jasmonate responsive AtWRKY genes, 22 were differentially expressed in at least two jasmonate treated datasets. *AtWRKY6*, *AtWRKY18*, *AtWRKY45*, and *AtWRKY53* were differentially expressed in three jasmonate treated datasets. Expression of *AtWRKY7*, *AtWRKY69* and *AtWRKY75* were significantly changed in response to jasmonate in four datasets. *AtWRKY40* and *AtWRKY47* expression were significantly changed in all five datasets. Notably, *AtWRKY47*, *AtWRKY69*, and *AtWRKY75* expression were significantly differentially regulated to jasmonate treatment in four or more microarray experiments, but did not survive application of the Benjamini-Hochberg false discovery (B-H FDR) in any dataset. Arabidopsis *WRKY6*, *WRKY11*, *WRKY17*, *WRKY25*, *WRKY46*, and *WRKY53*, which have known roles in jasmonate signaling, were identified as being differentially expressed, but did not survive the B-H FDR in any dataset (Additional file 2: Table S2). While the B-H FDR is less conservative than other procedures (e.g. Bonferroni correction), genes on the upper end of significant p-values (genes with small fold changes) may not be easily detected even if the response is consistent. This becomes apparent by the large reduction in significant genes after B-H FDR (Table 1). In total, eleven WRKYs survived the B-H FDR in at least one dataset indicating these are jasmonate responsive. We identified five WRKY TFs previously reported to be jasmonate responsive [27, 36, 38, 45]. Additionally, we identified at least six Arabidopsis WRKY TFs (*AtWRKY7*, *AtWRKY20*, *AtWRKY26*, *AtWRKY45*, *AtWRKY48*, and *AtWRKY72*) previously unreported to have a role in jasmonate response (Table 1 and Additional file 2: Table S2). Expression of *AtWRKY7*, *AtWRKY40*, and *AtWRKY45* changed significantly and survived the B-H FDR in two datasets. For these eleven AtWRKYs, the change in expression in response to jasmonate treatment was small, around 2.5 fold (Additional file 3: Table S3A-E). AtWRKY40 displayed the greatest change in expression, with a 9-fold induction of transcripts after 1 hr of treatment with MeJA. Collectively, at least 18% of AtWRKY (11 of 61), up to 64% (39 of 61) or more, were jasmonate responsive WRKYs based on the microarrays analyzed. Overall, at least 30% (22 of 72) of AtWRKY TFs play a role in jasmonate response indicating the importance of this family in the jasmonate signaling network. Further experiments may eventually reveal that upwards of 50% (39 of 72) of WRKYs are involved in Arabidopsis jasmonate response (Additional file 2: Table S2).

**Table 1:** **The Arabidopsis WRKY transcription factors differentially expressed in response to jasmonate treatment in five experiments before and after the application of the Benjamini-Hochberg false discovery rate**

			Genes after Two-Way ANOVA (P = 0.05)			Genes after Two-Way ANOVA (P = 0.05) and B-H FDR		
Dataset	Source	Samples used	No. genes	No. WRKY genes	WRKYs	No. genes	No. WRKY genes	WRKYs
E-ATMX-13	EMBL	MeJA treated timecourse (0.5, 2, and 6 hr) in cell suspension cultures	2819	11	WRKY6, WRKY9, WRKY15, WRKY18, WRKY25, WRKY26, WRKY39, WRKY40, WRKY47, WRKY54, WRKY69	116	none	none
E-GEOD-28600	EMBL	JA and JA + ABA treated (3 and 24 hr) T87 cell cultures	5279	21	WRKY1, WRKY6, WRKY7, WRKY16, WRKY21, WRKY35, WRKY36, WRKY38, WRKY40, WRKY43, WRKY45, WRKY47, WRKY52, WRKY53, WRKY54, WRKY67, WRKY69, WRKY70, WRKY71, WRKY72, WRKY75	533	3	WRKY7, WRKY38, WRKY70
E-MEXP-883	EMBL	MeJA treated (6 hr) WT and myc2 plants	3348	14	WRKY6, WRKY7, WRKY11, WRKY18, WRKY20, WRKY23, WRKY26, WRKY33, WRKY39, WRKY40, WRKY45, WRKY47, WRKY69, WRKY75	568	4	WRKY26, WRKY33, WRKY40, WRKY45
GSE21762	NCBI	JA treated WT and coi1 seedlings	3743	16	WRKY3, WRKY7, WRKY17, WRKY22, WRKY25, WRKY31, WRKY40, WRKY46, WRKY47, WRKY52, WRKY53, WRKY60, WRKY70, WRKY72, WRKY74, WRKY75	175	1	WRKY72
ME00337	TIAR	MeJA treated (0.5, 1, 3 hr) time course on WT seedlings	4796	15	WRKY3, WRKY7, WRKY18, WRKY20, WRKY21, WRKY23, WRKY38, WRKY40, WRKY45, WRKY47, WRKY48, WRKY53, WRKY60, WRKY69, WRKY75	950	6	WRKY7, WRKY18, WRKY20, WRKY40, WRKY45, WRKY48

Analysis was performed using a two-way ANOVA. Only WRKY genes responsive to jasmonate treatment are presented.

The small overlap between WRKY genes differentially expressed in response to jasmonate treatment in the microarray experiments suggested a tight developmental and/or spatiotemporal regulation in Arabidopsis. Two-way ANOVAs analyzing expression in response to time, as well as its combined effect with jasmonate treatment, further indicated AtWRKY regulation is temporally dependent (Additional file 4: Table S4A-C). The expression of 20 AtWRKY genes was time dependent (Additional file 4: Table S4A). Jasmonate treatment was found to regulate the expression of *AtWRKY38* and *AtWRKY70* in a time dependent manner. The genetic background of jasmonate signaling pathway mutants had less effect on WRKY gene expression. CORONATINE INSENSITIVE 1 (COI1) has been established as a jasmonate receptor [54, 55]. *AtWRKY72* was the only WRKY family member found to be regulated in a COI1-dependent manner when the B-H FDR was applied (Additional file 4: Table S4B). No WRKYs were found to be dependent on MYC2 (Additional file 4: Table S4C), a major transcriptional regulator of jasmonate signaling [56, 57]. These results indicate that response of Arabidopsis WRKY TFs to jasmonate treatment is highly dependent upon tissue, timing and culture conditions, and likely occurs through several major pathways. Furthermore, WRKY TFs may be important COI1-independent regulators of jasmonate response.

Unsupervised agglomerative hierarchical clustering analysis of AtWRKY TFs was performed to identify similar patterns of gene expression which may indicate related functions [58]. Gene expression of AtWRKYs formed two major clusters. Clustering of experiments revealed more similarities within an experiment than by jasmonate treatment (Additional file 5: Figure S1). Additionally, the two major clusters separated those experiments in which the sampled tissues were from either plants or cell cultures. These findings further support AtWRKY gene expression in response to jasmonate as highly dependent on culture conditions and environment. The two major clusters were further subdivided into two or three clusters. Jasmonate responsive WRKYs previously annotated or identified by our microarray analysis primarily occurred in cluster one and all of the three sub-clusters. The distribution of jasmonate responsive AtWRKYs indicates at least two major pathways for the regulation of AtWRKY gene expression. Interestingly, the only WRKY identified by microarray analysis to be COI1-depenedent, *AtWRKY72*, occurred in cluster 2b, distinct from expression patterns of other jasmonate responsive WRKYs. These data further suggest that there are complex tissue and environmental controls over jasmonate responses that likely occurs through several major pathways. These findings from Arabidopsis provide foundational information about the involvement of WRKY TFs in jasmonate response and for exploiting these factors in genetic engineering of transcriptional regulatory networks for natural product production.

### Identification of *Catharanthus*WRKY TFs

Previously we identified a MeJA responsive group III type WRKY TF, CrWRKY1, as important for the regulation of TIA in *Catharanthus* [46]. Furthermore, the Arabidopsis data indicates the AtWRKY family as important for regulating jasmonate response. Jasmonate responsive CrWRKY TF may be important for regulating the production of valuable TIAs. Elucidation of CrWRKYs regulating specialized metabolite production will be valuable for future genetic engineering projects to increase production of pharmaceutically valuable TIAs. As the first step to identify important CrWRKY regulators of specialized metabolism we sought to identify all WRKY family members in *Catharanthus*. The recent release of 14 medicinal plants Illumina sequenced transcriptomes by the Medicinal Plant Genomic Resource (MPGR), including *Catharanthus*, provides the opportunity to identify many CrWRKY TFs [59]. To identify CrWRKYs, we first downloaded all protein sequences from MPGR and isolated a single protein sequence for each locus. We assumed the individual copy of each locus reflects the total number of functional genes within the genome. While this method may include potential errors, such as RNA-sequencing artifacts, establishing single copies of genes allows the identification of WRKY family members and approximate family size. Due to possible variations in splicing and/or incomplete splicing of introns we searched contig assemblies with the longest predicted protein sequence for each gene in the MPGR database. All CrWRKY proteins identified from the MPGR database, described below, were manually verified to contain a WRKY domain. In several cases (CrWRKY8, CrWRKY13, CrWRKY17, CrWRKY21, CrWRKY34, CrWRKY37, and CrWRKY47), alignment results among *Catharanthus* contigs for a locus and the closest matching AtWRKY TF, were utilized to remove a conserved intron following the WRKYGQK consensus sequence or correct a frame shift, to generate a full WKRY domain sequence. As single base pair insertion in *CrWRKY8* was not clear by aligning other copies of this contig, the region spanning the insertion was cloned for verification.

Searching for the established invariant consensus sequences WRKYGQK and known alternative WRKYGKK, WRKYGEK, and WRKYGSK consensus sequences from the list of proteins, duplicate results were eliminated and 46 putative WRKY TFs were identified (Additional file 6: Table S5). Comparatively, MPGR annotated 47 potential CrWRKY TF encoding genes. However, only 35 WRKYs overlapped between manual searches for the consensus motifs and the MPGR annotated datasets.

To further validate the number of WRKY TFs a list of the single longest predicted proteins for each locus was submitted to the National Center for Biotechnology Information Conserved Domain Database (NCBI CDD) and the Samuel Roberts Nobel Foundation PlantTFcat (PlantTFcat) server, for protein domain identification [60]. The NCBI CDD identified 52 WRKY domain-containing proteins (Additional file 6: Table S5). Similarly, PlantTFcat (http://plantgrn.noble.org/PlantTFcat/) also identified 52 WRKY domain-containing proteins. The majority of additional proteins identified by NCBI CDD and PlantTFcat as WRKY TFs, had incomplete N-terminal ends of the WRKY domain (CrWRKY11, CrWRKY15, CrWRKY48, and CrWRKY49). One additional predicted WRKY TF, CrWRKY32, contained a WRKYGRK motif. CrWRKY9, which was identified by NCBI CDD, but not PlantTFcat, had an incomplete C-terminal portion of the WRKY domain. Contig Cra15757 was predicted by PlantTFcat to be a WRKY TF. Inspection of this protein sequence did not reveal the presence of a WRKY consensus or zinc finger binding motif. Of the 47 proteins annotated as WRKYs by MPGR, only 40 were found to be true WRKY TFs as identified by NCBI CDD and PlantTFcat. In total, 52 proteins in *Catharanthus* were predicted as WRKY TFs (Table 2).

**Table 2:** **A list of Catharanthus WRKY domain containing proteins along with locus number and group number are presented**

Catharanthus WRKY	Locus	Group
CrWRKY1	Cra16284	III
CrWRKY2	Cra549	I
CrWRKY3	Cra4234	I
CrWRKY4	Cra5497	I
CrWRKY5	Cra6088	I
CrWRKY6	Cra8145	I
CrWRKY7	Cra9152	I
CrWRKY8	Cra10348	I
CrWRKY9	Cra11128	I
CrWRKY10	Cra13321	I
CrWRKY11	Cra22691	I
CrWRKY12	Cra43671	I
CrWRKY13	Cra1311	IIa
CrWRKY14	Cra13263	IIa
CrWRKY15	Cra54213	IIa
CrWRKY16	Cra2068	IIb
CrWRKY17	Cra3503	IIb
CrWRKY18	Cra18915	IIb
CrWRKY19	Cra19580	IIb
CrWRKY20	Cra22725	IIb
CrWRKY21	Cra2271	IIc
CrWRKY22	Cra2950	IIc
CrWRKY23	Cra6519	IIc
CrWRKY24	Cra8670	IIc
CrWRKY25	Cra9369	IIc
CrWRKY26	Cra19330	IIc
CrWRKY27	Cra22395	IIc
CrWRKY28	Cra24943	IIc
CrWRKY29	Cra28262	IIc
CrWRKY30	Cra37309	IIc
CrWRKY31	Cra43896	IIc
CrWRKY32	Cra102390	IIc
CrWRKY33	Cra105225	IIc
CrWRKY34	Cra1702	IId
CrWRKY35	Cra3760	IId
CrWRKY36	Cra7867	IId
CrWRKY37	Cra17347	IId
CrWRKY38	Cra11684	IIe
CrWRKY39	Cra16307	IIe
CrWRKY40	Cra19395	IIe
CrWRKY41	Cra20290	IIe
CrWRKY42	Cra21821	IIe
CrWRKY43	Cra23742	IIe
CrWRKY44	Cra30069	IIe
CrWRKY45	Cra3799	III
CrWRKY46	Cra5093	III
CrWRKY47	Cra18989	III
CrWRKY48	Cra24719	III
CrWRKY49	Cra55720	
CrWRKY50	Cra56567	
CrWRKY51	Cra65443	
CrWRKY52	Cra70197	

Of the 52 possible WRKY TFs from *Catharanthus*, at least 48 appear to be authentic (Table 2). The MPGR database contained full WRKY domain sequences for 52 domains from 43 TFs. Partial domain sequences were found for nine WRKYs. 3′ rapid amplification of cDNA ends (RACE) or 5′ RACE was performed to obtain the necessary domain sequence for 5 WRKYs. 3′ RACE was performed on *CrWRKY9*. For *CrWRKY11*, *CrWRKY12*, *CrWRKY15*, and *CrWRKY48,* 5′ RACE was used to obtain the rest of the WRKY domain sequence. Clones could not be found for four genes (*CrWRKY49*, *CrWRKY50*, *CrWRKY51*, and *CrWRKY52*). Expression data, available from MPGR, revealed these four WRKYs are not present in any of the 23 samples sequenced. To validate the MPGR expression data, quantitative reverse transcription polymerase chain reaction (qRT-PCR) was used to measure the transcript levels of *CrWRKY49*, *CrWRKY50*, *CrWRKY51*, and *CrWRKY52*. Gene specific transcripts for *CrWRKY49*, *CrWRKY50*, *CrWRKY51*, and *CrWRKY52* could not be detected in root, stem, leaf, or whole plant samples. Transcripts for the same four WRKYs could also not be found in 0, 1, 2, and 4 hour MeJA-treated samples. This data suggests that these predicted partial WRKY sequences are not in any of our samples, and that they may be either artifacts of RNA-sequencing, temporally regulated, or induced by a factor not present in our growing conditions. WRKY TFs are known to play key roles in plant senescence [42]. However, senescing medicinal plant tissues were not utilized for sequencing in the MPGR. Inclusion of senescing tissues may slightly increase the total WRKY number to more closely reflect fully sequenced plant species. Future investigations with different treatment conditions may detect the expression of *CrWRKY49*, *CrWRKY50*, *CrWRKY51*, and *CrWRKY52*.

**Figure 1:**
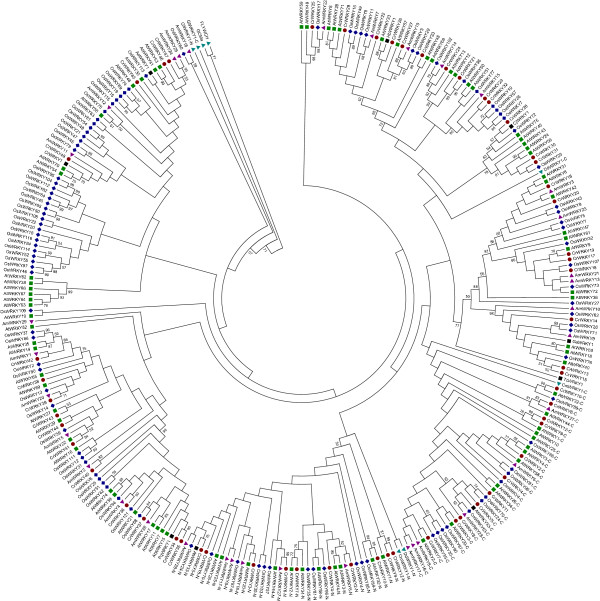
**The phylogenetic tree of** ***C. roseus*** **(red dot),** ***A. trichopoda*** **(inverted purple triangle),** ***A. thaliana*** **(green square), and** ***O. sativa*** **(blue diamond) was constructed using the Neighbor-Joining method with P-distance substitution model, pairwise-deletion, and a bootstrap value of 2000.** WRKY domain alignment was performed with ClustalW. Proteins used as an outgroup are indicated by a teal triangle. WRKY TFs from six additional medicinal species, *A. annua (*AaWRKY1*)*, *C. japonica (*CjWRKY1*)*, *G. arboreum (*GaWRKY1*)*, *H. brasiliensis (*HbWRKY1*)*, *P. quinquefolius (*PqWRKY1*)*, and *T. chinensis* (TcWRKY1), were included. WRKY TFs involved in regulating secondary metabolism are indicated with a black dot.

The *Catharanthus* WRKY family appears to be one of the smallest reported WRKY TF families to date. Only the moss *Physcomitrella patens*, the lycophyte *Selaginella moellendorffii*, and Castor bean (*Ricinus communis*), with 37, 35, and 47 WRKYs respectively, are reported to have fewer WRKY TFs [61]– [63]. Our results suggest that the *Catharanthus* WRKY family is similar in size to *Cucumis sativus*, *Fragaria vesca*, *Jatropha curcas*, and *Carica papaya* with 55, 56, 58 and 66 WRKYs, respectively [64]– [67]. To further investigate the size of the *Catharanthus* WRKY family, we identified the WRKY TFs from serpentwood (*Rauvolfia serpentina*) transcriptome sequences (Table 3) [59]. Serpentwood is closely related to *Catharanthus* and also produces pharmaceutically valuable TIAs (Additional file 7: Figure S2). We found 54 serpentwood TFs, a number close to the 52 WRKYs identified in *Catharanthus*. The number of WRKYs belonging to each subgroup was also similar between these two species (Additional file 8: Figure S3). However, as both serpentwood and *Catharanthus* WRKYs were identified from transcriptome data, the actual size of the families may be larger. To address this possibility, we identified WRKY families from tomato (*Solanum lycopersicum*) [68], potato (*Solanum tuberosum*) [69], pepper (*Capsicum annuum*) [70], and bladderwort (*Utricularia gibba*) [71], all species of which have complete genome sequence available (Table 3). We identified 81 WRKY TFs in tomato (Table 3, Additional file 9: Figure S4), as previously reported [72]. Bladderwort, pepper, and potato each contained 65, 66, and 75 complete WRKY TFs, respectively (Table 3, Additional file 8: Figure S3 and Additional file 9: Figure S4). These data suggest the ancestor of the Gentianales (*Catharanthus* and serpentwood), Lamiales (bladderwort), and Solanales (pepper, potato and tomato) likely contained around 65 WRKY TFs. Therefore, we conclude that greater than 75% (52 out of 65) of *Catharanthus* and serpentwood WRKY TFs were identified from transcriptome data. Together, the six asterid species contained a similar number of WRKY TFs as found in the Brassicales, Arabidopsis and papaya [66, 73]. These data, combined with that from other WRKY families [64]– [67], suggests that *Brachypodium distachyon* (86 WRKYs), *Oryza sativa* ssp. *japonica* (105 WRKYs), *Populus trichocarpa* (104 WRKYs), and *Zea mays* (119 WRKYs) may contain atypically large WRKY families [29, 33, 66, 74, 75] compared to other angiosperms. Arabidopsis and rice both contain expansions in the group III WRKY subfamily [29, 73], whereas an expansion of group IIe occurs in potato and tomato [72] (Table 3). We did not find any evidence for subfamily expansions in *Catharanthus* or serpentwood (Table 3; Figure 1).

**Table 3:** **The distribution of WRKY TFs from nine plant species**

Species	Complete WRKY TFs	Partial WRKY TFs	Group I	Group IIa	Group IIb	Group IIc	Group IId	Group IIe	Group III	Unassigned
*Amborella trichopoda*	29	3	7	2	4	5	2	4	5	3
*Arabidopsis thaliana*	72	2	14	3	8	18	7	8	14	2
*Capsicum annuum*	66	4	16	4	6	13	11	7	9	4
*Catharanthus roseus*	48	4	11	3	5	13	4	7	5	4
*Oryza sativa ssp. japonica*	93	6	15	4	8	16	7	10	34	5
*Rauvolfia serpentina*	49	5	10	2	4	12	5	5	5	11
*Solanum lycopersicum*	78	3	15	5	8	17	6	17	11	2
*Solanum tuberosum*	75	9	14	5	6	14	7	15	14	9
*Urticularia gibba*	65	7	16	4	4	18	7	11	5	7

WRKY TFs identified from the sequenced genomes of *A. trichopoda*, *A. thaliana*, *C. annuum*, *O. sativa*, *S. lycopersicum*, *S. tuberosum*, and *U. gibba. C. roseus* and *R. serpentina* WRKY TFs were identified from transcriptome sequences in the MPGR database. Complete and partial WRKY domain containing proteins were identified using the NCBI Conserved Domain Database. The presence of WRKY domains were manually verified and phylogenetic analyses were conducted to determine WRKY subgroups for each of the species.

### Phylogenetic analysis of *Catharanthus*WRKY TFs

To determine the relationship among *Catharanthus* WRKY TFs, a phylogenetic tree was constructed with 282 WRKY domains from 243 TFs from *Catharanthus*, *Amborella trichopoda*, Arabidopsis and rice (*Oryza sativa* ssp. *japonica*) (Figure 1). WRKY sequences from *Chlamydomonas reinhardtii* (XP_001692342), *Dictyostelium discoideum* (XP_643786), and *Giardia lamblia* (XP_001708807) were included as an outgroup. Additional, outgroup sequences include human GCMa (BAA13651) and FLYWCH CRAa (EAW85450). We used 84 and 105 WRKY domains from 72 and 94 TFs, from Arabidopsis and rice respectively, to construct the phylogenetic tree. Thirty-five domains from 29 *Amborella* WRKY TFs were also included in the phylogenetic analysis [76]. We incorporated the WRKY sequences of *Amborella*, an evolutionary basal angiosperm, to reduce long-branch attractions during phylogenetic tree construction. *Amborella* was selected over *Physcomitrella patens* (moss) and *Selaginella moellendorffii* (spikemoss) since the WRKY sequences from this phylogenetically important species remains unreported yet provides valuable insights about WRKY evolution. The phylogenetic tree contained 58 domains from 48 CrWRKY TFs (Additional file 10: Table S6). To ascertain potential functions, we compared *Catharanthus* and Arabidopsis WRKY TFs by identifying orthologs. We identified 11 group I, 32 group II, and five group III WRKY TFs in *Catharanthus*. Group II WRKY TFs can be classified into groups IIa, IIb, IIc, IId, or IIe [32]. In *Catharanthus,* we identified three group IIa, five group IIb, thirteen group IIc, four group IId and seven group IIe WRKY TFs.

Evolutionarily, group I WRKY TFs, such as those found in algae, are some of the most ancient of WRKYs [29, 73]. Recent evidence suggest that the group I WRKYs, and other WRKY TFs, originated from an ancestral group IIc-like domain [31]. As previously reported for this group, ten group I CrWRKYs contained two WRKY domains with the N-terminal domain forming a separate clade and the C-terminal WRKY domains forming part of the group IIc clade [29, 73]. To identify orthologs and paralogs in *Amborella*, Arabidopsis, *Catharanthus* and rice, we used OrthoMCL [77]. We found *Catharanthus* contains six coorthologs (CrWRKY2, CrWRKY3, CrWRKY4, CrWRKY5, CrWRKY8 and CrWRKY51) to AtWRKY33 (Table 4). According to the phylogenetic tree, CrWRKY5 is most closely related to AtWRKY33 (Figure 1).

**Table 4:** **Orthologs and paralogs for** *Arabidopsis* **and** *Catharanthus* **WRKY found using OrthoMCL**

*Catharanthus roseus*	*Arabidopsis thaliana*	Other
*CrWRKY1,* **CrWRKY48,** CrWRKY52	AtWRKY54, **AtWRKY70**	
CrWRKY2, CrWRKY3, CrWRKY4, **CrWRKY5, CrWRKY8,** CrWRKY51	AtWRKY20, *AtWRKY33*	CmWRKY1
CrWRKY6	AtWRKY44	
CrWRKY7	AtWRKY2	
CrWRKY9, CrWRKY12	AtWRKY1	
CrWRKY10	AtWRKY32	
**CrWRKY13,** CrWRKY14, CrWRKY15	**AtWRKY40**	*GaWRKY1*
CrWRKY16, CrWRKY20	**AtWRKY6,** AtWRKY31, AtWRKY42	*TcWRKY1*
CrWRKY17, **CrWRKY18**	**AtWRKY72**	
CrWRKY19	AtWRKY9	
**CrWRKY21,** CrWRKY22, **CrWRKY26,** CrWRKY27, CrWRKY29, CrWRKY33	**AtWRKY8,** AtWRKY12, AtWRKY23, **AtWRKY28, AtWRKY48, AtWRKY51,** AtWRKY57, AtWRKY71	*HbWRKY1*
CrWRKY23, CrWRKY32	**AtWRKY50**	
CrWRKY24	AtWRKY13	
CrWRKY25	AtWRKY49	
CrWRKY28	AtWRKY75	*CjWRKY1*
CrWRKY30, CrWRKY31	AtWRKY24, AtWRKY43, AtWRKY56	
CrWRKY34, **CrWRKY35**	**AtWRKY7**	*PqWRKY1*
**CrWRKY36**	AtWRKY21	
CrWRKY37	**AtWRKY11, AtWRKY17**	
**CrWRKY38**	AtWRKY69	
CrWRKY39	AtWRKY65	
**CrWRKY41,** CrWRKY43	AtWRKY22, AtWRKY27	
CrWRKY42	AtWRKY14, AtWRKY35	
**CrWRKY45,** CrWRKY46, CrWRKY47	AtWRKY41, AtWRKY46, **AtWRKY53**	*AaWRKY1*

WRKYs in ‘bold font’ are jasmonate responsive, either according to the literature or by our findings in this study. WRKY highlighted in italics are TFs known to regulate secondary metabolism in *A. thaliana*, *Artemisia annua*, *C. rosues*, *Coptis japonica*, *Gossypium arboreum*, *Hevea brasiliensis*, *Panax quinquefolius*, and *Taxus chinensis*.

Group IIa was the only group of WRKYs that had similar numbers between *Catharanthus* and Arabidopsis. Rice had four group IIa WRKY TFs whereas both Arabidopsis and *Catharanthus* each contained three. The three group IIa WRKYs from *Catharanthus* are coorthologs to AtWRKY40 (Table 4).

**Figure 2:**
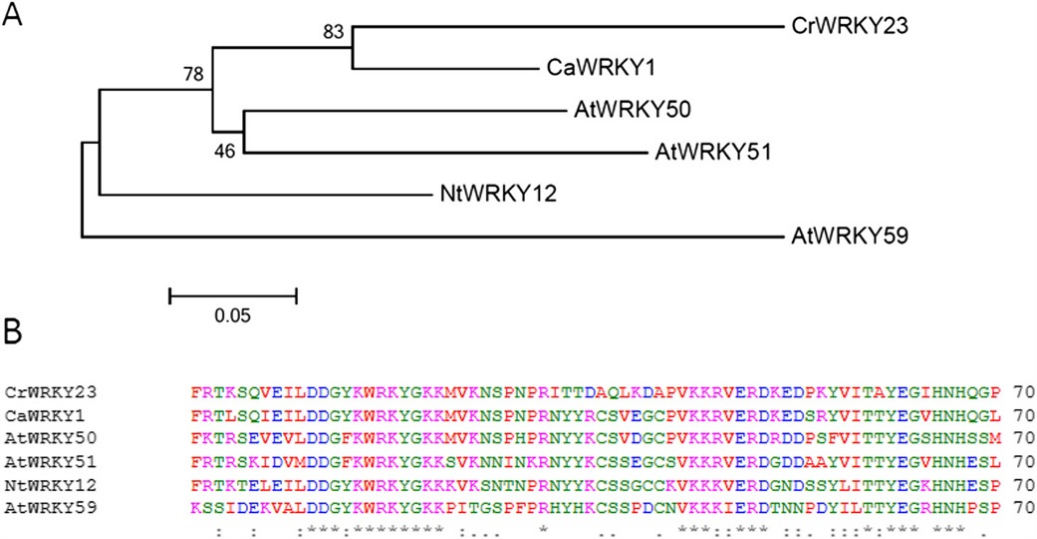
**The phylogenetic relationship and alignment of CrWRKY23 to other WRKYGKK containing WRKY transcription factors. A**. The phylogenetic tree was constructed in MEGA5 using the Neighbor-Joining method with P-distance substitution model, pairwise-deletion, and a bootstrap value of 2000. The tree is unrooted and branch lengths drawn to scale to evolutionary distances. Alignment was performed using ClustalW. **B**. Alignment of WRKY domain sequence was performed using ClustalW.

Previous reports indicate some plants contain variants of the highly conserved WRKYGQK domain, such as WRKYGKK, WRKYGEK, WRKYGSK, among others [29]. Variation in this region can reduce, eliminate, or alter DNA binding activity [78]. WRKY TFs with variants of the consensus sequence may recognize different *cis*-elements. We found AtWRKY50, AtWRKY51, and AtWRKY59 belong to the group IIc WRKY subfamily and possess a WRKYGKK motif as previously reported [32]. Two CrWRKYs were identified that contain variants of the highly conserved WRKYGQK motif. CrWRKY23 and CrWRKY32 contain WRKYGKK and WRKYGRK sequence motifs, respectively. Mutagenesis of the conserved glutamine was previously demonstrated to reduce, but not eliminate, DNA binding [78]. More recently, AtWRKY50 was found to generally bind the GAC core of the W-box with less preference for 5′ or 3′ bases [31]. Therefore, both WRKYGKK and WRKYGRK variants are expected to still bind DNA. *Nictotiana tabacum* WRKY12, containing a WRKYGKK motif, has been found to bind the WK-box *cis*-element (TTTTCCAC), but not the W-box, which regulates expression of the plant defense gene PATHOGENESIS RELATED1 [79]. However, *Capsicum annuum* WRKY1, a WRKYGKK motif WRKY TF involved in plant defense, can still recognize the W-box [80]. The phylogeny and protein alignment of the DNA-binding WRKY domains of CrWRKY23, CaWRKY1 and NtWRKY12 revealed more similarity of CrWRKY23 to CaWRKY1 (Figure 2A and B), suggesting that despite the variant WRKYGKK motif, CrWRKY23 likely still recognizes the W-box element or at least the GAC core. Although *Hordeum vulgare* WRKY46 (SUSIBA2) contains a WRKYGQK motif, HvWRKY46 recognizes the Sugar Responsive (AATAGAAAA) and W-box *cis*-elements to regulate barley genes involved in starch metabolism [81]. This leaves open the possibility that some *Catharanthus* WRKY TFs may not recognize W-box *cis*-elements.

Group III WRKY TFs are believed to have dramatically expanded during the evolution of angiosperms and can be classified into different subgroups depending on the species [34, 72]. Arabidopsis contains fourteen group III WRKY TFs which are further divided into eight group IIIa and six group IIIb. In Arabidopsis, most group III WRKY transcription are induced by plant pathogens [34]. We identified only five group III WRKY TFs in *Catharanthus*. Similarly, we identified 5 group III WRKYs in serpentwood and bladderwort (Table 3). Proportionally, the number of group III CrWRKY TFs is the smallest compared to Arabidopsis. The low number of group III CrWRKYs, and similar number from serpentwood and bladderwort, suggests this group has not undergone significant expansion such as occurred in rice or Arabidopsis [72]. CrWRKY1 and CrWRKY48 were found to be coorthologs to AtWRKY70 and AtWRKY54 (Table 4). AtWRKY70 modulates SA and jasmonate signaling [36]. Interestingly, CrWRKY1 differentially directs the flow of unknown precursors into TIA products [46], a feature possibly governed by its jasmonate responsive gene expression. CrWRKY45, CrWRKY46, and CrWRKY47 are coortholgs of AtWRKY41, AtWRKY46, and AtWRKY53. AtWRKY46 and AtWRKY53 are partially functionally redundant in regulating plant defense [43].

We previously reported the role of CrWRKY1 in regulating gene expression and TIA accumulation in *Catharanthus* [46]. CrWRKY1 is a group III WRKY with overall protein sequence homology closest to AtWRKY70, and corresponds to MPGR contig number Cra16284. Phylogenetically, CrWRKY1 is located towards the base of the group III clade and does not clearly group with its Arabidopsis or rice orthologs (Additional file 11: Figure S5A). To identify the unique feature of CrWRKY1, we analyzed the protein sequence alignment. The invariant tryptophan starting the WRKYGQK motif was used as the reference point for comparing alignments. Alignment of CrWRKY1 to other group III WRKY TFs revealed that CrWRKY1 lacks an amino acid between the two conserved cysteine residues at positions 21 and 29 (Additional file 11: Figure S5B). The closest rice WRKY TFs, OsWRKY21, OsWRKY61 and OsWRKY47, all have altered spacing within the WRKY domain sequence. OsWRKY47 possesses an additional proline residue between the WRKYGQK sequence and the conserved arginine residue at position 16 [32]. The conserved arginine at position 16 was changed to threonine followed by a TQS motif in OsWRKY61. OsWRKY47 contains an extra DDP sequence between positions 41 and 42 compared to all other Arabidopsis, rice, and *Catharanthus* group III WRKY TFs. The altered spacing in the WRKY domain may give these WRKY TFs unique structural properties important for target gene regulation.

### Expression profiling reveals multiple Jasmonate responsive CrWRKYs

MPGR provides RNA-sequencing based expression data from different tissues for all sequenced medicinal plants. For *Catharanthus*, RNA-sequencing data is also available for different tissues, seedlings, cell suspension cultures, and hairy root cultures. These data provide an opportunity to understand the induction of WRKY genes in response to conditions that induce the TIA pathway. Furthermore, several treatments allow for comparison of induction to the same hormone in varying tissues. In response to MeJA, a potent and important elicitor of natural product formation, including TIAs, in *Catharanthus* and other medicinal species [82]– [85], MPGR expression data indicates multiple WRKY TFs are either up or down regulated in *Catharanthus*.

**Figure 3:**
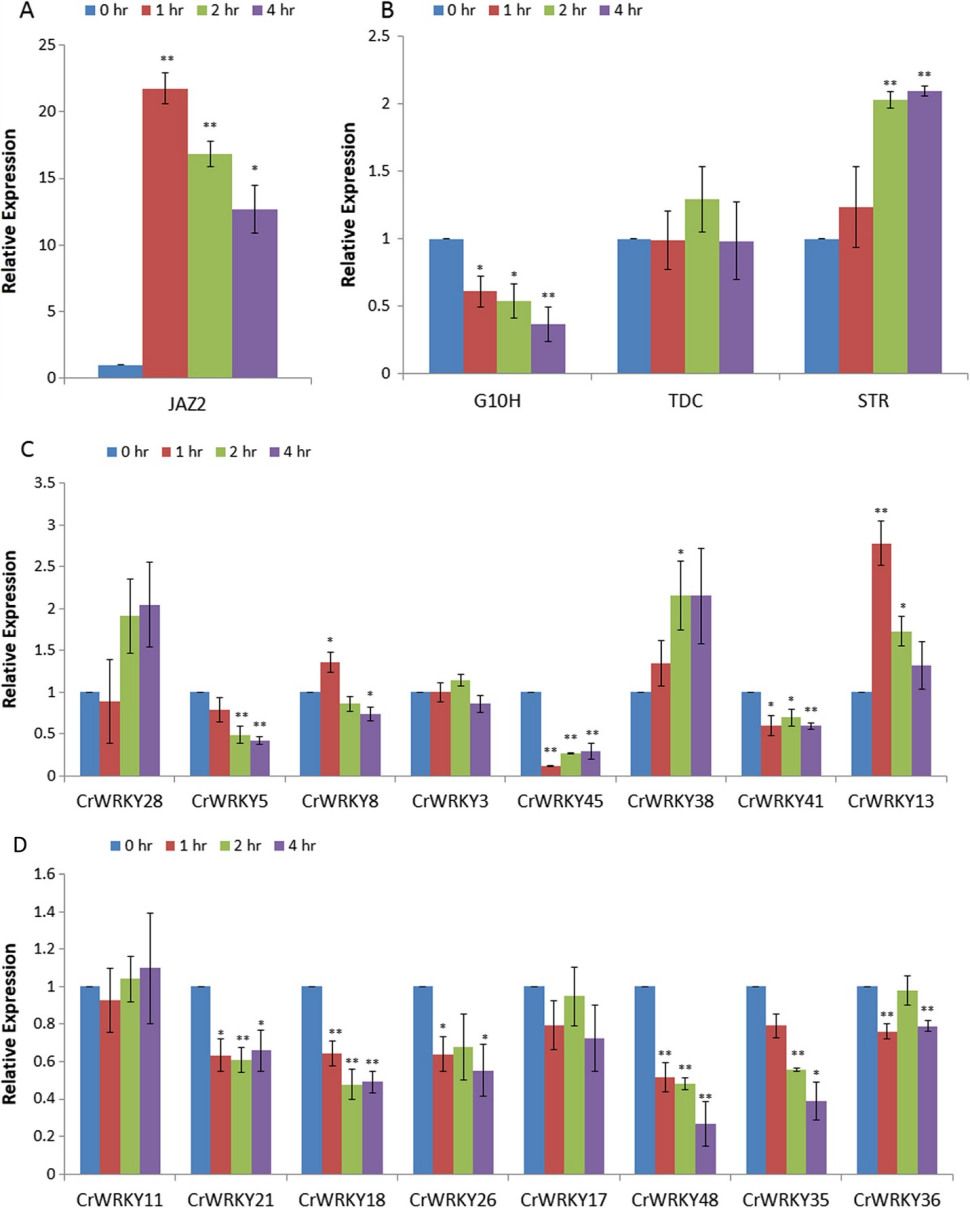
**Quantitative reverse-transcription PCR (qRT-PCR) for quantification of gene expression was performed on mature** ***Catharanthus*** **plants.** Three whole plants were combined for each biological replicate. Each time point consisted of three biological replicates. Three technical replicates were measured per time point sample. **A**. Expression of *Catharanthus JAZ2* transcripts were determined in after 0, 1, 2, or 4 hours of MeJA treatment. **B**. Expression of the TIA biosynthetic genes *G10H*, *TDC*, and *STR*. C-D. Expression of 16 *Catharanthus* WRKY transcription factors in response to 0, 1, 2, and 4 hours of MeJA treatment. Significant and highly significant, p-value < 0.05 or 0.01 respectively, changes in gene expression were determined using a Student’s T-test.

To identify or validate WRKY TFs that are up- or down regulated by MeJA, we performed qRT-PCR on whole plant samples (root, stem, and leaves) that were collected from one month old soil grown plants at 0, 1, 2, and 4 hours after MeJA treatment. Successful induction with MeJA was verified by measuring *JAZ2* expression (Figure 3A). To determine which WRKY TFs were possible regulators of TIA production, we sought to measure the expression of multiple pathway genes such as *G10H*, *TDC*, and *STR* (Figure 3B). These genes were selected to represent early (*G10H* and *TDC*) and middle portions (STR) of the TIA pathway. Any WRKY induced prior or simultaneously to these genes possibly could regulate that corresponding portion of the pathway and any subsequent segments.

We selected at least two genes from each CrWRKY subgroup. Four genes (*CrWRKY5*, *CrWRKY8*, *CrWRKY13*, and *CrWRKY28*) were selected based on involvement of their orthologs in regulating secondary metabolism genes in other species. Analysis of sixteen CrWRKYs identified twelve which displayed significant changes in expression in response to jasmonate (Figure 3C-D). The fold change for most CrWRKYs with a significant response to MeJA was 2 fold or less (Figure 3C-D), similar to our microarray findings for jasmonate responsive Arabidopsis WRKYs (Additional file 3: Table S3). *CrWRKY8* was up-regulated 1 hour after MeJA treatment then decreased by 4 hours after treatment. *CrWRKY5* was down-regulated significantly at both 2 and 4 hour after MeJA treatment. *CrWRKY13*, similar to the ABA responsive *AtWRKY40*, was significantly up-regulated 1 and 2 hours after MeJA treatment. *CrWRKY38* was up-regulated by 2 hours after MeJA treatment. *CrWRKY18*, *CrWRKY21*, *CrWRKY41*, *CrWRKY45*, and *CrWRKY48* were all significantly down-regulated at all time points after MeJA treatment. Two WRKYs, *CrWRKY26* and *CrWRKY36*, had a bimodal expression pattern that was down-regulated at 1 and 4 hours, but not 2 hours, after MeJA treatment. A bimodal expression pattern has been observed for some regulators of the TIA pathway [15]. *CrWRKY35* was down-regulated 2 and 4 hours after treatment. Nine of 12 CrWRKYs analyzed were down-regulated to MeJA treatment. In total at least 25% (12 of 48), and probably more, of CrWRKY TFs are regulated by jasmonate. Of the twelve jasmonate responsive CrWRKYs, nine have an AtWRKYs ortholog which were either previously reported and/or identified here by microarray analysis (p < 0.05 and survived B-H FDR) as differentially regulated by jasmonate (Figure 3C-D, Additional file 2: Table S2). When compared to the less stringent list of AtWRKYs which had expression significantly changed (p < 0.05) in response to jasmonate, but did not survive the B-H FDR, all twelve CrWRKYs have orthologs to jasmonate responsive AtWRKYs.

To identify potential WRKYs regulating TIA biosynthesis through the jasmonate signaling pathway, we compared the induction times of WRKY genes (Figure 3C-D) to early and mid- biosynthetic genes of the pathway (Figure 3B). Similar to previous reports in cell cultures [15], induction of *STR* by MeJA began approximately 2 hours after treatment. However, expression of *G10H* decreased starting at 1 hour after MeJA treatment, and was further down-regulated 4 hours after treatment (Figure 3B). *TDC* transcript levels remained unchanged to MeJA treatment in mature *Catharanthus* plants. Prior reports of *TDC* [7, 13, 86]– [88] and *G10H* [13, 86]– [88] transcript induction by jasmonate treatment was identified in seedlings, hairy roots, or cell cultures; however, our experiments were performed in intact mature *Catharanthus* plants. Expressions of WRKY TFs possibly contributing to TIA regulation are predicted to be altered before early and mid steps of the TIA pathway. Expression of *CrWRKY8*, *CrWRKY13*, *CrWRKY18*, *CrWRKY21*, *CrWRKY26*, *CrWRKY36*, *CrWRKY41*, *CrWRKY45* and *CrWRKY48* changed by 1 hour after MeJA treatment indicating these WRKYs could possibly regulate the expression of early TIA pathway genes. Altered expression of all twelve CrWRKYs responding to MeJA occurred by 2 hours after treatment, the same time at which significant induction of *STR* occurred. Contrary to *TDC* and *G10H*, which contain four and one W-boxes in their promoters respectively [46, 89], the characterized *STR* promoter does not contain any W-box elements for WRKYs to bind, but this does not exclude the possibility that WRKY regulate other TFs controlling *STR* expression. The spatio-temporal regulation of CrWRKYs, by reducing *TDC* responsiveness and down-regulating *G10H*, is one possible reason why mature *Catharanthus* plants do not accumulate TIA in response to jasmonate treatment [90]. These findings suggest that all CrWRKYs we ascertained as differentially expressed in response to jasmonate are possible regulators of early and middle steps of TIA biosynthesis. Presumably, these CrWRKY could also regulate downstream steps of the pathways which are temporally expressed later.

### The Jasmonate Response of *Catharanthus*WRKY Varies Among Plant Culture Conditions

**Figure 4:**
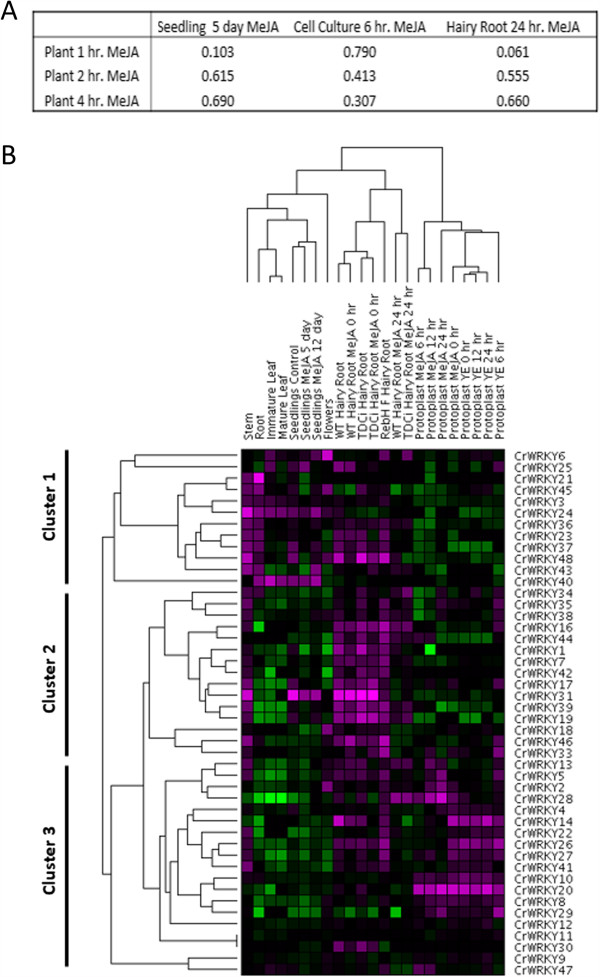
**Pearson correlation analysis of WRKY expression data and hierarchical clustering of MPGR transcriptome data for the CrWRKY TF family. A**. Pearson correlation between fold change of qRT-PCR expression and MPGR datasets for MeJA treated samples. Fold change for both sets was calculated using the reference gene EF1α as an internal control. **B**. Hierarchical cluster analysis of MPGR transcriptome data for the CrWRKY TF family was performed using GenePattern. The clustering method was a pairwise average linkage with distance measured using the Pearson correlation coefficient. Data was log transformed. The median value was subtracted from each row. Color is based on global expression with purple being up-regulated and green down-regulated.

We sought to determine the similarities between our qRT-PCR results and the transcriptome data published by MPGR. As we used one month-old plants to quantify gene expression, and no data on MeJA treated mature plants are provided by MPGR, we correlated our data to three different datasets each representing one aspect of our samples (5 day MeJA treated seedlings, 6 hour MeJA treated cell suspension cultures, and 24 hour MeJA treated hairy root cultures). Seedlings treated with MeJA most closely represent our samples in physiology as both are whole plant tissues; however, the MPGR dataset used seedlings rather than mature plants, which may respond to MeJA differently [90]. While cell cultures are considerably different in physiology from whole plants, the earliest time sample (6 hours after MeJA treatment) was closest to our sample times of 1, 2, and 4 hours after MeJA treatment. Hairy root cultures require several weeks to develop to sufficient size; therefore, the age of this tissue most likely represents a similar age as our plant samples, despite our shorter MeJA treatment time. The Pearson correlation coefficient was calculated to measure the relationship between the datasets. *CrWRKY11* and *CrWRKY21* were excluded from the correlations as they appear two times in the MPGR datasets without expression values. The correlation between MPGR seedling and cell culture datasets, as well as between cell culture and hairy root datasets, was quite low (r = 0.179 and r = 0.227 respectively), indicating considerable difference in CrWRKY response to MeJA in cell culture systems. However, there was a high correlation between seedling and hairy root MPGR datasets (r = 0.892) for MeJA treated CrWRKY genes. Our qRT-PCR data showed that the correlations ranged from 0.061, between 1 hour MeJA treated plants and 24 hour MeJA treated hairy roots, to 0.790, between 1 hour MeJA treated plants and 6 hour MeJA treated cell suspension cultures (Figure 4A). Overall, the three MPGR datasets correlated well with all three time points of qRT-PCR data (median value of the 9 correlations = 0.555) indicating similar expression changes in response to jasmonate treatment. Increasing time after MeJA treatment in whole plants increased the correlation with seedling and hairy root cultures. Cell cultures, despite higher similarities in the 4 hour MeJA treated plant and 6 hour MeJA treated cell culture time frame, showed a lower correlation between the 1 hour MeJA treated plant and 6 hour MeJA treated cell cultures. Similar to Arabidopsis, these findings in *Catharanthus* suggest significant differences exist between jasmonate response in various cultural conditions, including intact seedlings, adult plants, cell cultures, and hairy root cultures.

Gene expression clusters often contain genes with related functions [58], including those in natural product formation [91]. Recently, clustering of MPGR expression data has aided the identification of *Catharanthus* IRIDOID SYNTHASE [92]. To identify potential clusters of CrWRKY TFs with similar expression pattern which may indicate WRKY functions, we performed a hierarchical clustering. Unsupervised agglomerative hierarchical clustering of 23 transcriptome gene expression datasets from MPGR revealed three primary clusters: a plant tissue cluster, a hairy root cluster, and a protoplast cluster (Figure 4B). Clusters of plant culture type indicate a greater difference between cultural conditions than between MeJA treatment. However, clear differences exist between MeJA treated and untreated samples within subgroups of each primary cluster. Clustering of CrWRKY gene expression revealed three primary clusters. CrWRKYs of cluster one were most up-regulated in different plant tissues, suggesting a role in plant development. The second cluster of WRKY genes is up-regulated in hairy root cultures. Members of cluster two may be important for regulating metabolism and resource direction into primarily root produced alkaloids, such as ajmalicine and serpentine. Identification of *CrWRKY1*, which plays a role in serpentine production, in cluster two supports this idea. *CrWRKY46*, ortholog to *AaWRKY1*, a trichome expressed WRKY in *Artemisia annua*, was also found in this cluster. *CrWRKY34* and *CrWRKY35* orthologous to PqWRKY1 which is suggested to regulate terpene biosynthesis in *Panax quinquefolius* (American ginseng) roots also occur in this cluster. The third cluster consisted of CrWRKY that were up-regulated in response to MeJA or yeast extract (YE), an elicitor of TIA biosynthesis, in protoplasts. Most of this cluster was also up-regulated in hairy root cultures. Most CrWRKYs orthologous to WRKYs regulating natural product formation in other species were identified in this cluster. Importantly, four CrWRKYs (*CrWRKY2*, *CrWRKY5*, *CrWRKY13*, and *CrWRKY28*), similar to those with known roles in secondary metabolism (Table 4), were identified as part of the same sub-cluster in cluster three. The four members of this cluster may play key roles in regulation of natural product formation in *Catharanthus*. A second sub-cluster of cluster three, composed of six members, contained five CrWRKYs (*CrWRKY4*, *CrWRKY14*, *CrWRKY22*, *CrWRKY26*, and *CrWRKY27*) which are orthologs to WRKY TFs regulating natural products in other species. The sixth member of this sub-cluster, *CrWRKY41*, is also a jasmonate responsive WRKY. Members of this sub-cluster may also be important for regulation of natural products in *Catharanthus*. CrWRKY TFs, determined by qRT-PCR to be MeJA responsive, were distributed across all three clusters, indicating jasmonate broadly regulates WRKYs from each cluster.

### Predicted role of CrWRKY orthologs in secondary metabolism

*Catharanthus* produces alkaloids, terpenes and latex, all classes of compounds that contain biosynthetic genes involved in their production which have been implicated to be regulated by WRKY TFs in other species [48, 49, 51]. To associate biosynthesis of natural compounds with jasmonate-responsive CrWRKYs, we compared *Catharanthus* WRKY TFs to those known to control secondary metabolism in other plant species (Table 4). CjWRKY1 is involved in the regulation of the benzylisoquinoline alkaloid berberine [48]. In *Catharanthus*, CrWRKY28 grouped closely with CjWRKY1 (Figure 1). The ortholog of CjWRKY1 in Arabidopsis is AtWRKY75.

AtWRKY33 plays a role in regulating biosynthesis of camalexin, an indole ring and N-containing defense molecule, and functions downstream of MITOGEN-ACTIVATE PROTEIN KINASE 3 and 6 [52]. Recently, CrMPK3 was shown to regulate TIA accumulation [93]. As *Catharanthus* produces over 130 different TIA metabolites, the multiple coorthologs to AtWRKY33 may be important for regulating diverse products of this pathway. However, further experiments are needed to demonstrate whether the orthologous TFs in *Catharanthus* act downstream of CrMPK3 and are involved in TIA biosynthesis. Interestingly, CmWRKY1, from *Chlamydomonas*, was also an ortholog to AtWRKY33, suggesting a possible early function of this TF in defense and regulating secondary metabolism.

AaWRKY1, which is involved in regulating the accumulation of artemisinin, has three coortholgs in *Catharanthus*, CrWRKY45, CrWRKY46, and CrWRKY47 (Table 4). In Arabidopsis three group IIIa WRKYs, AtWKRY41, AtWRKY46, and AtWRKY53 are coortholgs to AaWRKY1. In *Catharanthus*, increased production of HMGR and terpenes have negatives effect on the accumulation of certain TIAs [94]. In *A. annua*, AaWRKY1 affects the expression of 3-HYDROXY-3-METHYLGLUTARYL-COA REDUCTASE (HMGR) [50], a rate limiting enzyme in the mevalonate pathway. Both AaWRKY1 and CrWRKY46 are jasmonate responsive genes; therefore, at least CrWRKY46 may have an evolutionarily conserved function in regulating the flux of carbon into *Catharanthus* terpenes.

The rate limiting enzyme in the production of paclitaxel, DBAT, is regulated by TcWRKY1 [47]. Phylogenetically, TcWRKY1 is basal to the group IIa and IIb clades (Figure 1). *Catharanthus* CrWRKY16 and CrWRKY20 were identified as coorthologs to TcWRKY1 (Table 4). In Arabidopsis, the group IIb AtWRKY6, AtWRKY31, and AtWRKY42 were found to be coortholgs to TcWRKY1 (Table 4).

GaWRKY1, from cotton, regulates a sesquiterpene cyclase leading to the production of gossypol [49]. AtWRKY40 was found to be the Arabidopsis ortholog to GaWRKY1 (Table 4). AtWRKY18 and AtWRKY60 formed their own ortholog group independent of AtWRKY40. *Catharanthus,* however, contains three coortholgs to GaWRKY1, CrWRKY13, CrWRKY14, and CrWRKY15. As with AtWRKY40, these CrWRKYs may have a role in negative regulation of ABA response [95] and positive regulation of jasmonate responses [38]. Supporting this idea, like AtWRKY40 and GaWRKY1, we found expression of CrWRKY13 was induced by jasmonate treatment. Drought, salinity, and cold all affect TIA accumulation in *Catharanthus* [12, 96], thus at least CrWRKY13 may function in regulating the accumulation of TIAs in response to abiotic stress and plant defense.

*HbWKRY1* expression is related to latex production in rubber trees [51]. In *Catharanthus*, CrWRKY21, CrWRKY22, CrWRKY26, CrWRKY27, CrWRKY29, and CrWRKY33 are coorthologs to HbWRKY1. Six coothrologs (AtWRKY8, AtWRKY12, AtWRKY23, AtWRKY28, AtWRKY48, AtWRKY51, and AtWRKY71) to HbWRKY1 exist in Arabidopsis (Table 4). Like HbWRKY1, at least four Arabidopsis (AtWRKY8, AtWRKY28, AtWRKY48, and AtWRKY51) and two *Catharanthus* WRKYs (CrWRKY21 and CrWRKY26) are regulated by jasmonate. Phylogenetically, the group IIc AtWRKY23 and CrWRKY26 are the WRKYs most similar to HbWRKY1 in Arabidopsis and *Catharanthus,* respectively (Figure 1). As *Catharanthus* also produces a latex compound, the jasmonate regulated CrWRKY26 or one of the other paralogs, may function in the regulation of latex or terpene production in *Catharanthus*.

Heterologous over-expression, in Arabidopsis, of the MeJA responsive American ginseng WRKY TF, *PqWRKY1*, increased drought and salt stress tolerance, in addition to regulating terpene biosynthetic genes [53]. AtWRKY7 in Arabidopsis and CrWRKY34 and CrWRKY35 in *Catharanthus* are orthologs to PqWRKY1 (Table 4). Contrary to the report by Sun et al. [53], which classifies PqWRKY1 as a group IIc WRKY, we found PqWRKY1 actually falls within the IId subgroup when compared to the entire Arabidopsis WRKY family (Figure 1). AtWRKY7, CrWRKY35, and PqWRKY1 are each regulated by jasmonate supporting the possible conserved evolutionary function of these proteins in regulating terpene biosynthesis.

Kalde et al. [34] reported a role of most Arabidopsis group III WRKY TFs in plant defense. Overall, no clear trend was observed for WRKY TFs possibly involved in secondary metabolism belonging to a specific group or subgroup. Further work is needed to verify the predicted roles of these CrWRKYs in the regulation of secondary metabolism.

Comparative genetics across species has provided invaluable information that lead to the isolation and functional understanding of several key regulators in natural product formation. In Arabidopsis, the bHLH factor AtMYC2 is known as a central regulator of jasmonate signaling pathway. The orthologs of *AtMYC2*, *CrMYC2* and *NtMYC2*, from *Catharanthus* and tobacco, respectively, have thus been isolated and characterized. While Arabidopsis does not produce TIAs or nicotine, CrMYC2 and NtMYC2 act in the jasmonte signaling pathway to regulate biosynthesis of these metabolites [16, 97]. Moreover, AtMYC2 can bind the jasmonate-responsive elements present in the promoter of *Catharanthus ORCA3,* an AP2/EFR TF gene, and activates its expression, illustrating the conserved nature of these orthologous regulators [98]. These reports further strengthen our reasoning for cross-species comparison of WRKY TFs from *Catharanthus*, Arabidopsis, and other medicinal plant to identify regulators conserved in jasmonate response and possibly secondary metabolite production.

**Figure 5:**
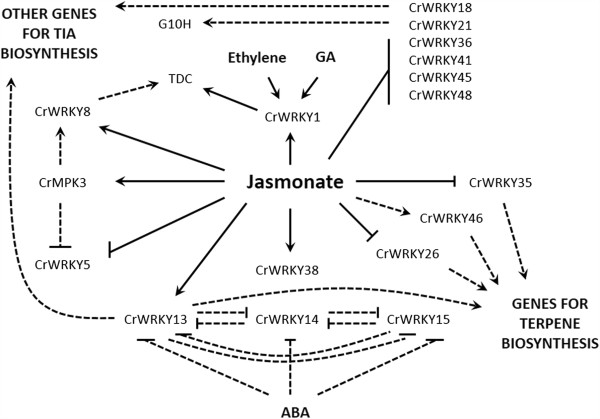
**A model of WRKY TFs function in** ***Catharanthus*** **based on expression data and known roles in** ***A. thaliana.*** The model depicts CrWRKYs which were either similar to a WRKY with a known role in regulating natural product formation in another species or had transcript level differentially expressed in response to MeJA treatment. Jasmonate has both positive and negative effects on CrWRKY transcript accumulation which is possibly important for fine-tuning TIA and terpene biosynthetic gene expression. The hormones abscisic acid (ABA), ethylene, and gibberellin (GA) also are likely important for regulation of *Catharanthus* WRKYs. Solid lines depict known regulations and dashed lines indicate hypothetical regulatory interactions.

Comparison of CrWRKYs with orthologs from other species, that are known to regulate natural products or respond to jasmonate treatment, helped us develop a model for WRKY regulation of TIA biosynthesis in *Catharanthus* (Figure 5). In this model, jasmonate acts as a central regulator of the TIA pathway with both positive and negative effects on WRKYs. Phytohormones, including ABA, ethylene and gibberellin (GA), are also likely involved in CrWRKYs regulation. Overall, this work provides a fundamental base for which future experiments can be designed to help elucidate the molecular mechanisms controlling the biosynthesis of highly valuable TIAs.

## Conclusion

Taken together, our results illustrate a role for the Arabidopsis WRKY family in regulating jasmonate response. These findings strengthened our reasoning for investigating *Catharanthus* jasmonate responsive WRKY TF which are potentially involved in regulation of TIA biosynthesis. Results from Arabidopsis and *Catharanthus* suggest that the regulation of WRKY gene expression in response to jasmonate is dependent upon environmental and spatio-temporal context. Such information can be important in designing metabolic engineering projects. Furthermore, we identified numerous jasmonate responsive orthologs between AtWRKY and CrWRKY TFs that may be functionally conserved or partially conserved. The jasmonate responsive CrWRKYs are potential candidate TFs for having key roles in modulating jasmonate signaling and regulating TIA biosynthesis. Information on how AtWRKYs response to various phytohormones and stresses may also apply to *Catharanthus*. This information may be useful for understanding how other phytohormones also contribute to the regulation of TIA production. Moreover, elucidation of CrWRKY functions may provide valuable insights into the regulation of natural product biosynthesis in other medicinal plants.

## Methods

### Plant growth conditions

*Catharanthus* ‘Little Bright Eyes’ seeds were surface sterilized and were germinated in the dark at 30°C for 3 days on MS plates, before being then transferred to an ambient temperature 24 h light photoperiod tissue culture room for an additional 4 days. Seedlings were transferred to soil and grown at ambient temperature under 24 h light. Samples were collected from 1 month-old *Catharanthus* plants treated with MeJA for 0, 1, 2, or 4 hours. The MeJA experiment was performed once with each time having three replicates. Three plants were combined from each replicate time sample. MeJA treatment consisted of spray application of 100 μM MeJA then placing plants under a clear plastic dome sealed with tape. Whole plants were harvested, roots quickly washed, and then frozen in liquid nitrogen.

### WRKY TF Identification

Contigs translated into protein sequence were downloaded from the MPGR. The single longest copy of each contig translated into protein sequence was identified using Microsoft Excel. Each unique contig number, which translated into some protein sequence, was determined to represent a unique gene distinguished by locus number. To differentiate potential WRKY genes each distinct contig locus number, but not different length variants of the same locus number, were considered as a unique product. As observed in Arabidopsis and other species, the multiple contig copies that comprise many of the loci may represent splice variants, not fully sequenced transcripts or different alleles. A Microsoft Excel file containing all protein encoding contigs was searched to manually identify WRKY and WRKYGQK invariant motif containing proteins. A FASTA file of the single longest protein encoding contig for the entire genome was submitted to the NCBI CDD and PlantTFcat servers to identify whole and partial WRKY domains containing contigs. The process was performed for *Amborella trichopoda*, *Arabidopsis thaliana*, *Capsicum annuum*, *Catharanthus roseus*, *Oryza sativa* ssp. *japonica*, *Rauvolfia serpentina*, *Solanum lycopersicum, Solanum tuberosum*, and *Urticularia gibba*. A file containing WRKY TFs from *Catharanthus*, *Amborella*, Arabidopsis, and rice was submitted to OrthoMCL [77] to identify orthologs and paralogs. WRKY TFs involved in regulating secondary metabolism from other species were also included. GenBank accession numbers for medicinal plant WRKY TFs included are: *AaWRKY1* (FJ390842), *CjWRKY1* (AB267401), *TcWRKY1* (JQ250831), *GaWRKY1* (AY507929), *HbWRKY1* (GU372969), and *PqWRKY1* (AEQ29014).

### Phylogenetic tree construction

The unrooted phylogenetic trees for *Catharanthus*, *Amborella*, Arabidopsis, and *Oryza sativa* ssp. *japonica* and medicinal plant WRKY TFs were constructed using the MEGA5 software. The neighborhood joining method, with bootstrap values of 2000, was utilized to conduct the phylogeny test. The analysis used p-distance of amino acid sequence to determine substitution rate. Gaps or missing data were excluded as needed, according to the pairwise deletion option. Phylogenetic trees analyzing the bladderwort, pepper, potato, serpentwood, and tomato WRKY families were constructed in the same way.

### RNA extraction

RNA was extracted using an extraction buffer composed of 1% 1,5-naphthalenedisulfonic acid and 4% p-aminosalicylic acid prepared in diethylpryocarbonate (DEPC) treated water. A 5 M sodium hydroxide solution was added until the extraction buffer was fully dissolved. For each RNA sample 5 mL of extraction buffer solution was mixed with 5 mL of liquefied phenol. Ground samples were added to the extraction buffer/phenol solution, vortexed 1 minute, 5 mL of chloroform added, then vortexed again. Samples were spun down for 10 minutes at 6000 rpm at 4°C. The aqueous phase was transferred to a 50 mL centrifuge tube and 1/10^th^ the volume of 3 M sodium acetate (pH 5.3) was added along with 2 times the volume of chilled 100% ethanol. Samples were incubated on ice 1 hr prior to centrifugation. The supernatant was discarded and the pellet dried for 30 minutes. The dried pellet was resuspended in 4 mL of autoclaved DEPC treated water and 2.5 mL of 8 M lithium chloride and incubated at 4°C overnight. The RNA was then precipitated by centrifuging at the above. The pellet was rinsed with chilled DEPC treated 80% ethanol. The ethanol was decanted and the RNA allowed to dry for 30 minutes before resuspending in sterile DEPC treated water.

### cDNA Synthesis and gene expression

Synthesis of first strand cDNA from total RNA isolated from plant tissue and quantitative reverse transcription polymerase chain reaction (qRT-PCR) were performed as previously reported [89]. Samples for the MeJA treatment consisted of 3 biological replicates each with 3 technical replicates. The comparative cycle threshold method was used to measure the transcript levels. All primers used for qRT-PCR can be found in Additional file 12: Table S7. Significant differences in gene expression were calculated using the Student’s T-test. P-values of 0.05 and 0.01 were considered significant and highly significant, respectively.

### 5′ and 3′ rapid amplification of cDNA Ends (RACE) and cloning

5′ and 3′ RACE was performed using the RACE kit (Invitrogen) as directed by the manufacturer. A nested set of PCRs was performed to isolate the target sequence. The first PCR reaction used the AAP primer and a gene specific primer; whereas the second nested reaction used the AUAP primer and a second gene specific primer. 3′ RACE was performed as for gene expression cDNA synthesis with the modification of using the 3′ AP primer to create a 3′ adapter. The 3′ target sequence was amplified through PCR using a nested set of gene specific primers and the adapter specific 3′ AUAP primer. All 3′ and 5′ RACE primers are listed in Additional file 13: Table S8.

### Cloning and sequencing of partial WRKY domains

Partial WRKY domain sequences to be cloned were amplified using 5′ or 3′ RACE. The sequence was then separated on an agarose gel and the DNA purified using a Wizard® SV Gel and PCR Clean-Up System (Promega). The purified DNA was ligated into pGEM-T Easy vector (Promega). Plasmid isolation was performed using a Wizard® Plus SV Minipreps DNA Purification System (Promega). 250–300 ng plasmid DNA were sequenced with either the T7 or SP6 primer using DTCS Quick Start (Beckmann) according to the manufacturer’s protocol. Sequencing was performed with a CEQ™ 8000 Genetic Analyzer System (Beckman Coulter) Sanger sequencer.

### Arabidopsis microarray analysis and gene expression

Jasmonate treated microarray datasets were collected from NCBI, EMBL, and TAIR. RMA Express was used for array normalization of each experiment [99]. Background adjustment, quantile normalization, and median polish were applied. Data was exported as log transformed data then analyzed by two-way ANOVA using the MEV software [100]. Two-way ANOVAs were performed on each dataset to determine response to jasmonate treatment and another variable (genotype or time). Controls and probes not linked to a gene were eliminated post-ANOVA prior to application of the false discovery rate. The B-H FDR was calculated in Microsoft Excel according to Thissen et al. [95]. Significant differences (p < 0.05) were determined before and after application of the Benjamini-Hochberg false discovery rate (B-H FDR). Significant differences before the B-H FDR was applied were both included because qRT-PCR for CrWRKY TFs indicated small (less than 2 fold), yet significant, changes to jasmonate treatment.

### Hierarchical clustering and correlations

The Pearson correlation coefficient was calculated to measure the relationship between qRT-PCR and MPGR datasets. First, the fold change for *Catharanthus* WRKY genes from the MPGR dataset was calculated in reference to 0 hour control treatments. Differences between control and MeJA treated datasets were then adjusted using the *Catharanthus* reference gene EF1α (Cra3894) as an internal control [13], as this gene was used as the internal control for qRT-PCR expression measurements. The correlation coefficient between fold changes in expression was calculated using Microsoft Excel.

Unsupervised agglomerative hierarchical clustering was performed using the GenePattern [96] website (http://genepattern.broadinstitute.org). The Pearson correlation was used as a distance measure for both row and column clustering. The clustering method was pairwise-average linkage with a row centering. Row centering was performed by subtracting the median value of each row. A global color scheme using a color gradient was applied for visualization. Purple, black, and green indicate increased gene expression, no change and decreased gene expression respectively. For Arabidopsis microarray data expression values were first analyzed with RMA Express and log transformed values exported for analysis with GenePattern [93, 96].

## Availability of supporting data

*Catharanthus* and serpentwood protein sequences used in this project was obtained from the MPGR database (http://medicinalplantgenomics.msu.edu/index.shtml). Sequences for Arabidopsis proteins were obtained from TAIR10 (http://www.arabidopsis.org/). Protein sequences from *Amborella* (http://www.amborella.org/), bladderwort (http://genomevolution.org/CoGe/OrganismView.pl?oid=36222), pepper (http://peppergenome.snu.ac.kr/), and rice (http://rice.plantbiology.msu.edu/) and were obtained from their respective databases. Tomato and potato protein sequences were obtained from the Sol Genomics Network database (http://solgenomics.net/). Sequence alignments and phylogenetic trees generated by this study were deposited into TreeBase under study identification number 15861 (http://purl.org/phylo/treebase/phylows/study/TB2:S15861).

## Electronic supplementary material

Additional file 1: Table S1: The list of WRKY TFs present or absent from the Affymetrix arrays used in this study. The Arabidopsis Affymetrix array contains probes to identify the expression of 61 WRKY TFs. Eleven of the 72 WRKY TFs in Arabidopsis are not represented on the array. (DOCX 12 KB)

Additional file 2: Table S2: The WRKY TFs identified as having significantly altered gene expression in at least one jasmonate treated dataset. WRKYs cited as identified in this study are those which were had significantly altered gene expression and survived the B-H FDR in at least one dataset. N/A indicates a probe to identify that WRKY is not available on the Affymetrix array but has been reported to be involved in jasmonate response. References are shown for WRKYs with reported function in jasmonate response. (DOCX 14 KB)

Additional file 3: Table S3: The fold change of jasmonate responsive Arabidopsis WRKY TFs from five microarray datasets. Only those jasmonate responsive AtWRKYs which survived application of the B-H FDR are included. (DOCX 54 KB)

Additional file 4: Table S4: Arabidopsis WRKY TFs were analyzed for differential expression by genotype in A) *coi1* and B) *myc2* mutants or C) by time. Analysis was performed using a two-way ANOVA. WRKYs before and after the application of the B-H FDR are presented. (DOCX 65 KB)

Additional file 5: Figure S1: Hierarchical cluster analysis of the Arabidopsis WRKY TF family was performed using GenePattern. The clustering method was a pairwise average linkage with distance measured using the Pearson correlation coefficient. Data was log transformed. The median value was subtracted from each row. Color is based on global expression with purple being up-regulated and green down-regulated. (TIFF 7 MB)

Additional file 6: Table S5: WRKY domain containing proteins were identified using 4 sources: manual searching, PlantTFcat, NCBI CDD, and MPGR. Rows indicate overlap in genes identified from the different sources. The bottom row provides a total number of genes identified by each method. (DOCX 15 KB)

Additional file 7: Figure S2: A phylogenetic tree constructed with nine plant species. The species tree was computed using the NCBI Common Tree then visualized with MEGA5 software. (TIFF 214 KB)

Additional file 8: Figure S3: The phylogenetic tree with *A. thaliana* (green square), *A. trichopoda* (purple triangle), *C. annuum* (teal dot), *O. sativa* (blue diamond), *R. serpentina* (red dot), and *U. gibba* (gold square) was constructed in MEGA5 using the Neighbor-Joining method with P-distance substitution model and a bootstrap value of 2000. Proteins used as an outgroup are indicated by a black triangle. WRKY domain alignment was performed with ClustalW. (PDF 841 KB)

Additional file 9: Figure S4: The phylogenetic tree with *A. thaliana* (green square), *A. trichopoda* (purple triangle), *O. sativa* (blue diamond), *S. lycopersicum* (gold square), and S. tuberosum (red dot) was constructed in MEGA5 using the Neighbor-Joining method with P-distance substitution model and a bootstrap value of 2000. Proteins used as an outgroup are indicated by a black triangle. WRKY domain alignment was performed with ClustalW. (PDF 691 KB)

Additional file 10: Table S6: *Catharanthus* contained at least 48 WRKY TFs with 56 WRKY domains. The 70 amino acid sequence of each WRKY domain is provided. (DOCX 22 KB)

Additional file 11: Figure S5: A-B. A. The phylogenetic relationship and alignment of CrWRKY1 to other group III WRKY TFs. The phylogenetic tree was constructed in MEGA5 using the Neighbor-Joining method with P-distance substitution model and a bootstrap value of 2000. WRKY domain alignment was performed with ClustalW. B. Alignment of the closest related rice WRKY genes and *Catharanthus* group III WRKYs was performed using ClustalW. (TIFF 11 MB)

Additional file 12: Table S7: A list of primers used in qRT-PCR to measure transcript levels. (DOCX 14 KB)

Additional file 13: Table S8: A list or primers used in cloning to isolate full WRKY domains for those TFs in the *Catharanthus* MPGR database with partial domain sequences. (DOCX 15 KB)

## Abbreviations

MPGR: Medicinal Plant Genomics Resource
PCR: Polymerase chain reaction
qRT-PCR: Quantitative reverse-transcription polymerase chain reaction
TF: Transcription factor
TIA: Terpene indole alkaloid.
