# Supplementary material for: Analyses of Catharanthus roseus and Arabidopsis thaliana WRKY transcription factors reveal involvement in jasmonate signaling
Source: BMC Genomics. 2014 Jun 20;15(1):502. doi: 10.1186/1471-2164-15-502 (PMC4099484; doi:10.1186/1471-2164-15-502)
Supplement: Supplementary file 1 — Additional file 1: Table S1: The list of WRKY TFs present or absent from the Affymetrix arrays used in this study. The Arabidopsis Affymetrix array contains probes to identify the expression of 61 WRKY TFs. Eleven of the 72 WRKY TFs in Arabidopsis are not represented on the array. (DOCX 12 KB) [file 12864_2013_6239_MOESM1_ESM.docx]

**Supplemental Table 1. The list of WRKY TFs present or absent from the Affymetrix arrays used in this study.**

| **Present on AFFY Arrays** | **Absent from AFFY Arrays** |
| --- | --- |
| WRKY1, WRKY2, WRKY3, WRKY4, WRKY6, WRKY7, WRKY8, WRKY9, WRKY10, WRKY11, WRKY12, WRKY13, WRKY14, WRKY15, WRKY16, WRKY17, WRKY18, WRKY19, WRKY20, WRKY21, WRKY22, WRKY23, WRKY25, WRKY26, WRKY27, WRKY28, WRKY30, WRKY31, WRKY32, WRKY33, WRKY34, WRKY35, WRKY36, WRKY38, WRKY39, WRKY40, WRKY42, WRKY43, WRKY44, WRKY45, WRKY46, WRKY47, WRKY48, WRKY52, WRKY53, WRKY54, WRKY55, WRKY56, WRKY57, WRKY58, WRKY60, WRKY61, WRKY65, WRKY66, WRKY67, WRKY69, WRKY70, WRKY71, WRKY72, WRKY74, WRKY75 | WRKY24, WRKY29, WRKY41, WRKY49, WRKY50, WRKY51, WRKY59, WRKY62, WRKY63, WRKY64, WRKY68 |
|  |  |

The *Arabidopsis* Affymetrix array contains probes to identify the expression of 61 WRKY TFs. Eleven of the 72 WRKY TFs in *Arabidopsis* are not represented on the array.
