# Supplementary material for: Analyses of Catharanthus roseus and Arabidopsis thaliana WRKY transcription factors reveal involvement in jasmonate signaling
Source: BMC Genomics. 2014 Jun 20;15(1):502. doi: 10.1186/1471-2164-15-502 (PMC4099484; doi:10.1186/1471-2164-15-502)
Supplement: Supplementary file 2 — Additional file 2: Table S2: The WRKY TFs identified as having significantly altered gene expression in at least one jasmonate treated dataset. WRKYs cited as identified in this study are those which were had significantly altered gene expression and survived the B-H FDR in at least one dataset. N/A indicates a probe to identify that WRKY is not available on the Affymetrix array but has been reported to be involved in jasmonate response. References are shown for WRKYs with reported function in jasmonate response. (DOCX 14 KB) [file 12864_2013_6239_MOESM2_ESM.docx]

**Supplemental Table 2. The WRKY TFs identified as having significantly altered gene expression in at least one jasmonate treated dataset.**

| **JA Responsive WRKY** | **No. Datasets Expression Changed** | **Reference** |
| --- | --- | --- |
| WRKY1 | 1 |  |
| WRKY3 | 2 |  |
| WRKY6 | 3 | [1] |
| WRKY7 | 4 | This study |
| WRKY8 | 0 | [2] |
| WRKY9 | 1 |  |
| WRKY11 | 1 | [3] |
| WRKY15 | 1 |  |
| WRKY16 | 1 |  |
| WRKY17 | 1 | [3] |
| WRKY18 | 3 | This study, [4] |
| WRKY20 | 2 | This study |
| WRKY21 | 2 |  |
| WRKY22 | 1 |  |
| WRKY23 | 2 |  |
| WRKY25 | 2 | [5] |
| WRKY26 | 2 | This study |
| WRKY28 | 0 | [6] |
| WRKY31 | 1 |  |
| WRKY33 | 1 | This study, [7,8] |
| WRKY35 | 1 |  |
| WRKY36 | 1 |  |
| WRKY38 | 2 | This study, [9] |
| WRKY39 | 2 |  |
| WRKY40 | 5 | This study, [4] |
| WRKY43 | 1 |  |
| WRKY45 | 3 | This study |
| WRKY46 | 1 |  |
| WRKY47 | 5 |  |
| WRKY48 | 1 | This study |
| WRKY50 | N/A | [10] |
| WRKY51 | N/A | [10] |
| WRKY52 | 2 |  |
| WRKY53 | 3 | [11] |
| WRKY54 | 2 |  |
| WRKY60 | 2 |  |
| WRKY62 | N/A | [12] |
| WRKY67 | 1 |  |
| WRKY69 | 4 |  |
| WRKY70 | 2 | This study, [13,14] |
| WRKY71 | 1 |  |
| WRKY72 | 2 | This study |
| WRKY74 | 1 |  |
| WRKY75 | 4 |  |

WRKYs cited as identified in this study are those which were had significantly altered gene expression and survived the B-H FDR in at least one dataset. N/A indicates a probe to identify that WRKY is not available on the Affymetrix array but has been reported to be involved in jasmonate response. References are shown for WRKYs with reported function in jasmonate response.
