# Supplementary material for: Analyses of Catharanthus roseus and Arabidopsis thaliana WRKY transcription factors reveal involvement in jasmonate signaling
Source: BMC Genomics. 2014 Jun 20;15(1):502. doi: 10.1186/1471-2164-15-502 (PMC4099484; doi:10.1186/1471-2164-15-502)
Supplement: Supplementary file 3 — Additional file 3: Table S3: The fold change of jasmonate responsive Arabidopsis WRKY TFs from five microarray datasets. Only those jasmonate responsive AtWRKYs which survived application of the B-H FDR are included. (DOCX 54 KB) [file 12864_2013_6239_MOESM3_ESM.docx]

**Supplemental Table 3. The fold change of jasmonate responsive Arabidopsis WRKY TFs from five microarray datasets.**


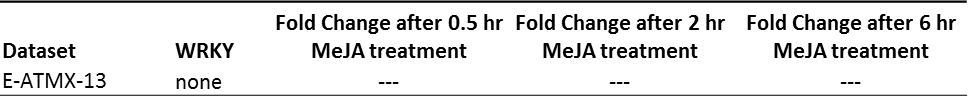

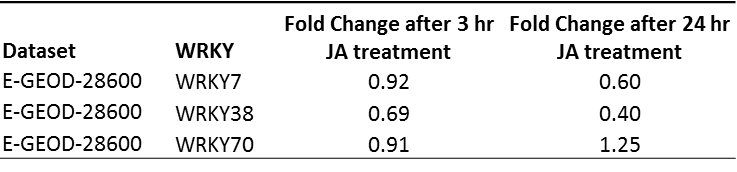

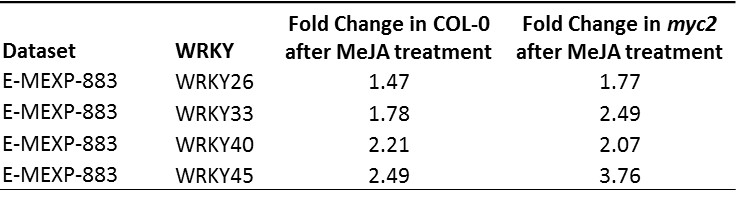

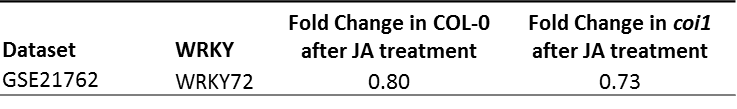


A.

B.

C.

D.

E.

Only those jasmonate responsive AtWRKYs which survived application of the B-H FDR are included.
