# Supplementary material for: Analyses of Catharanthus roseus and Arabidopsis thaliana WRKY transcription factors reveal involvement in jasmonate signaling
Source: BMC Genomics. 2014 Jun 20;15(1):502. doi: 10.1186/1471-2164-15-502 (PMC4099484; doi:10.1186/1471-2164-15-502)
Supplement: Supplementary file 4 — Additional file 4: Table S4: Arabidopsis WRKY TFs were analyzed for differential expression by genotype in A) coi1 and B) myc2 mutants or C) by time. Analysis was performed using a two-way ANOVA. WRKYs before and after the application of the B-H FDR are presented. (DOCX 65 KB) [file 12864_2013_6239_MOESM4_ESM.docx]

**Supplemental Table 4. *Arabidopsis* WRKY TFs were analyzed for differential expression by genotype in A) *coi1* and B) *myc2* mutants or C) by time.**


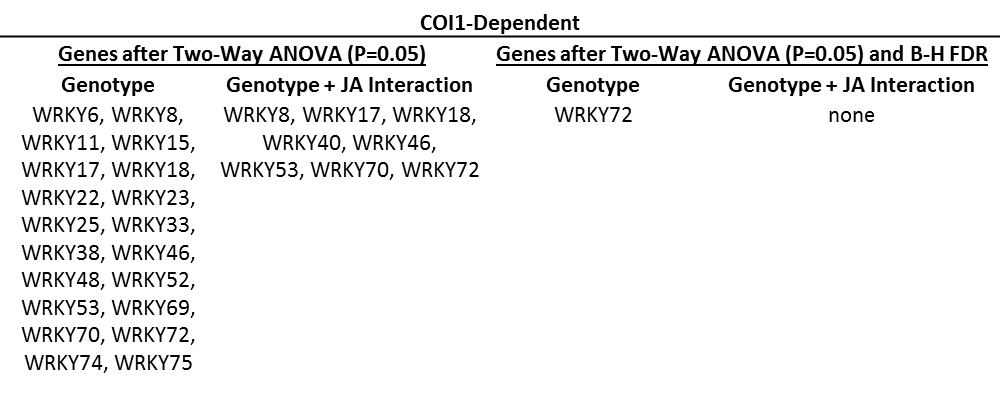

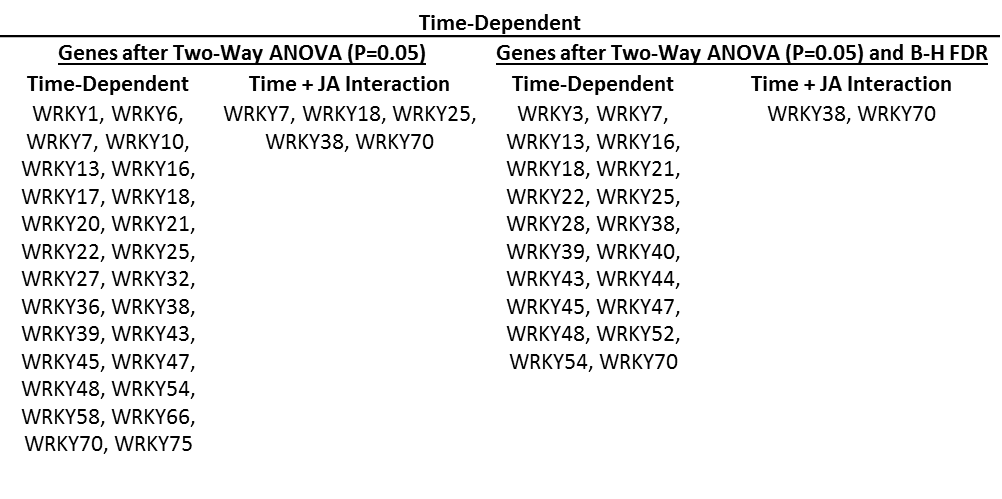


B

A


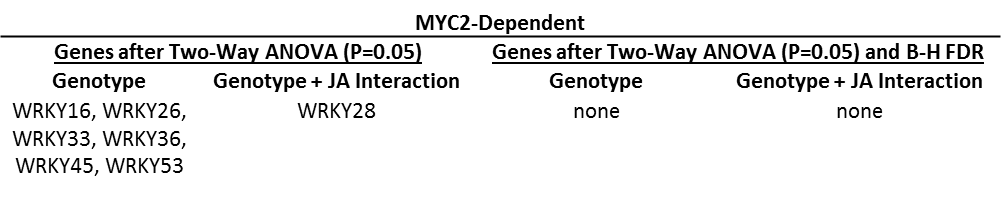


C


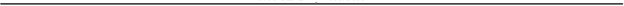


Analysis was performed using a two-way ANOVA. WRKYs before and after the application of the B-H FDR are presented.
