# Supplementary figures and images for: Analyses of Catharanthus roseus and Arabidopsis thaliana WRKY transcription factors reveal involvement in jasmonate signaling
Source: BMC Genomics. 2014 Jun 20;15(1):502. doi: 10.1186/1471-2164-15-502 (PMC4099484; doi:10.1186/1471-2164-15-502)

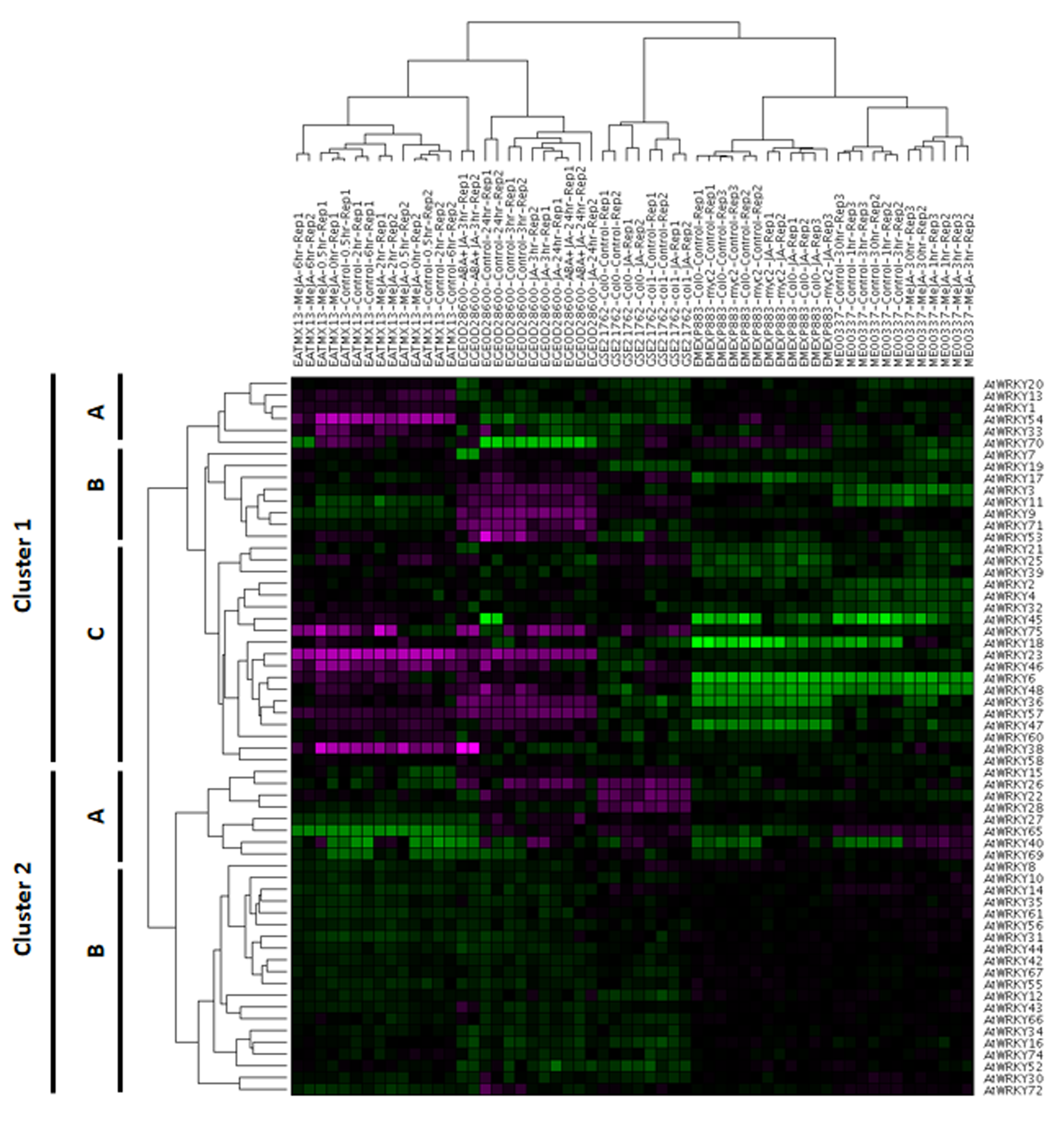

Supplement: Supplementary file 5 — Additional file 5: Figure S1: Hierarchical cluster analysis of the Arabidopsis WRKY TF family was performed using GenePattern. The clustering method was a pairwise average linkage with distance measured using the Pearson correlation coefficient. Data was log transformed. The median value was subtracted from each row. Color is based on global expression with purple being up-regulated and green down-regulated. (TIFF 7 MB) [file 12864_2013_6239_MOESM5_ESM.tiff]

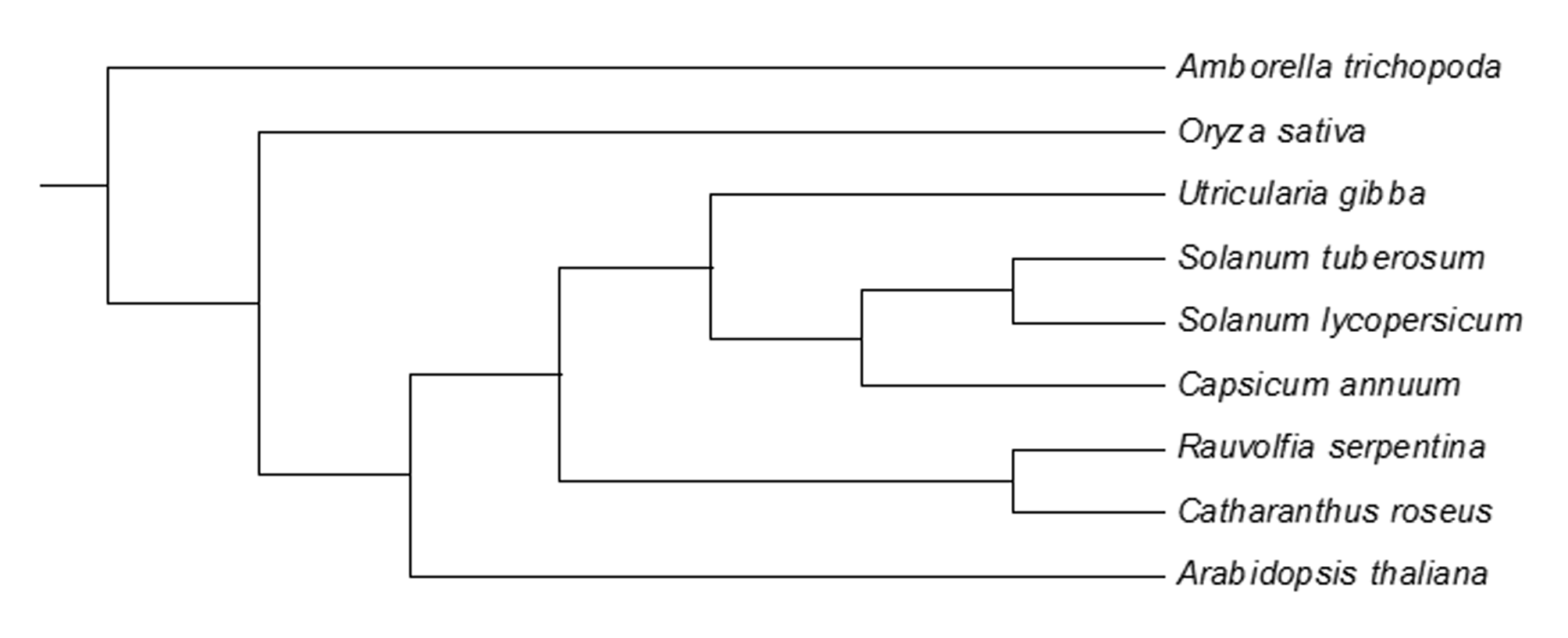

Supplement: Supplementary file 7 — Additional file 7: Figure S2: A phylogenetic tree constructed with nine plant species. The species tree was computed using the NCBI Common Tree then visualized with MEGA5 software. (TIFF 214 KB) [file 12864_2013_6239_MOESM7_ESM.tiff]

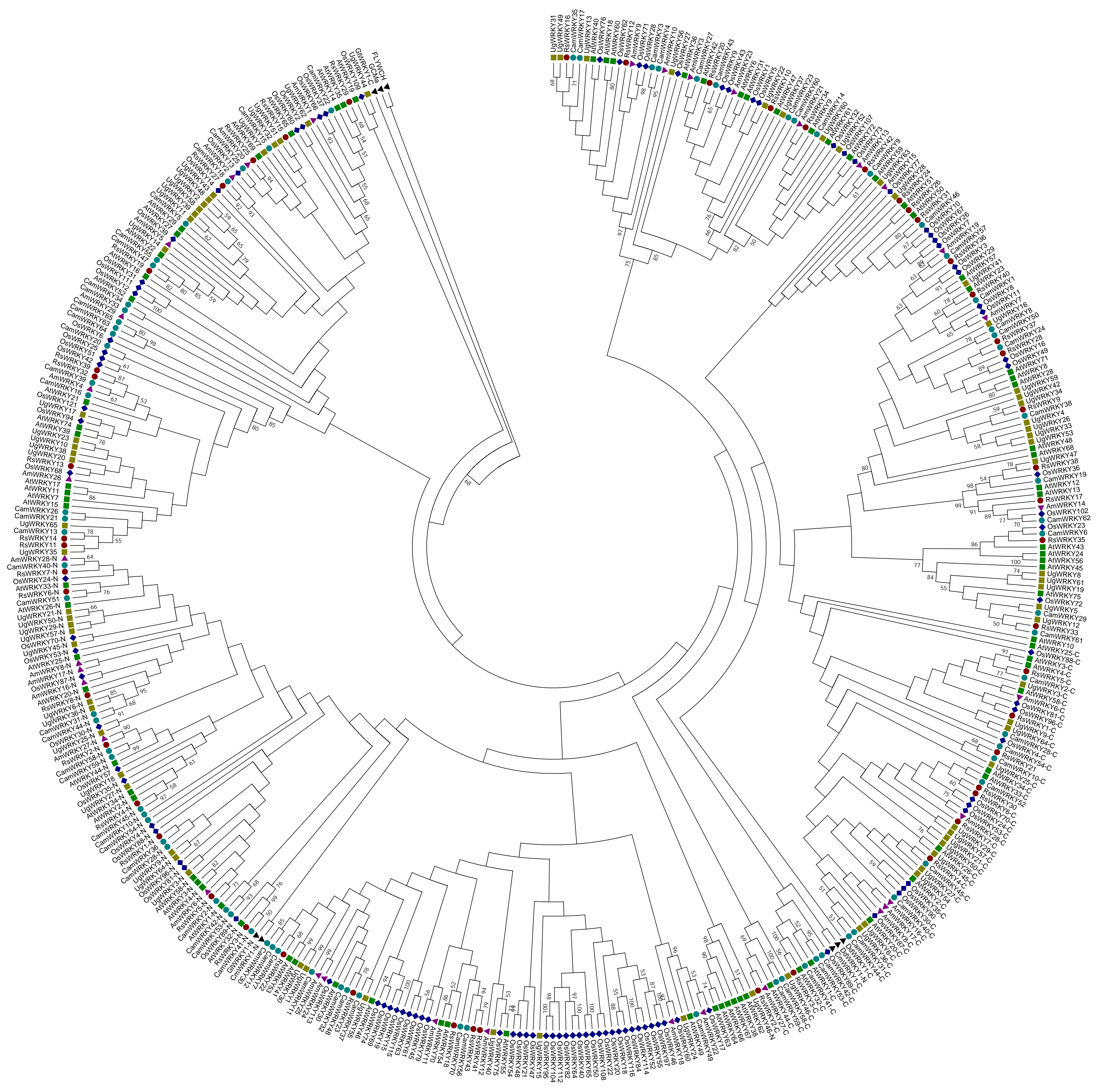

Supplement: Supplementary file 8 — Additional file 8: Figure S3: The phylogenetic tree with A. thaliana (green square), A. trichopoda (purple triangle), C. annuum (teal dot), O. sativa (blue diamond), R. serpentina (red dot), and U. gibba (gold square) was constructed in MEGA5 using the Neighbor-Joining method with P-distance substitution model and a bootstrap value of 2000. Proteins used as an outgroup are indicated by a black triangle. WRKY domain alignment was performed with ClustalW. (PDF 841 KB) [file 12864_2013_6239_MOESM8_ESM.pdf]

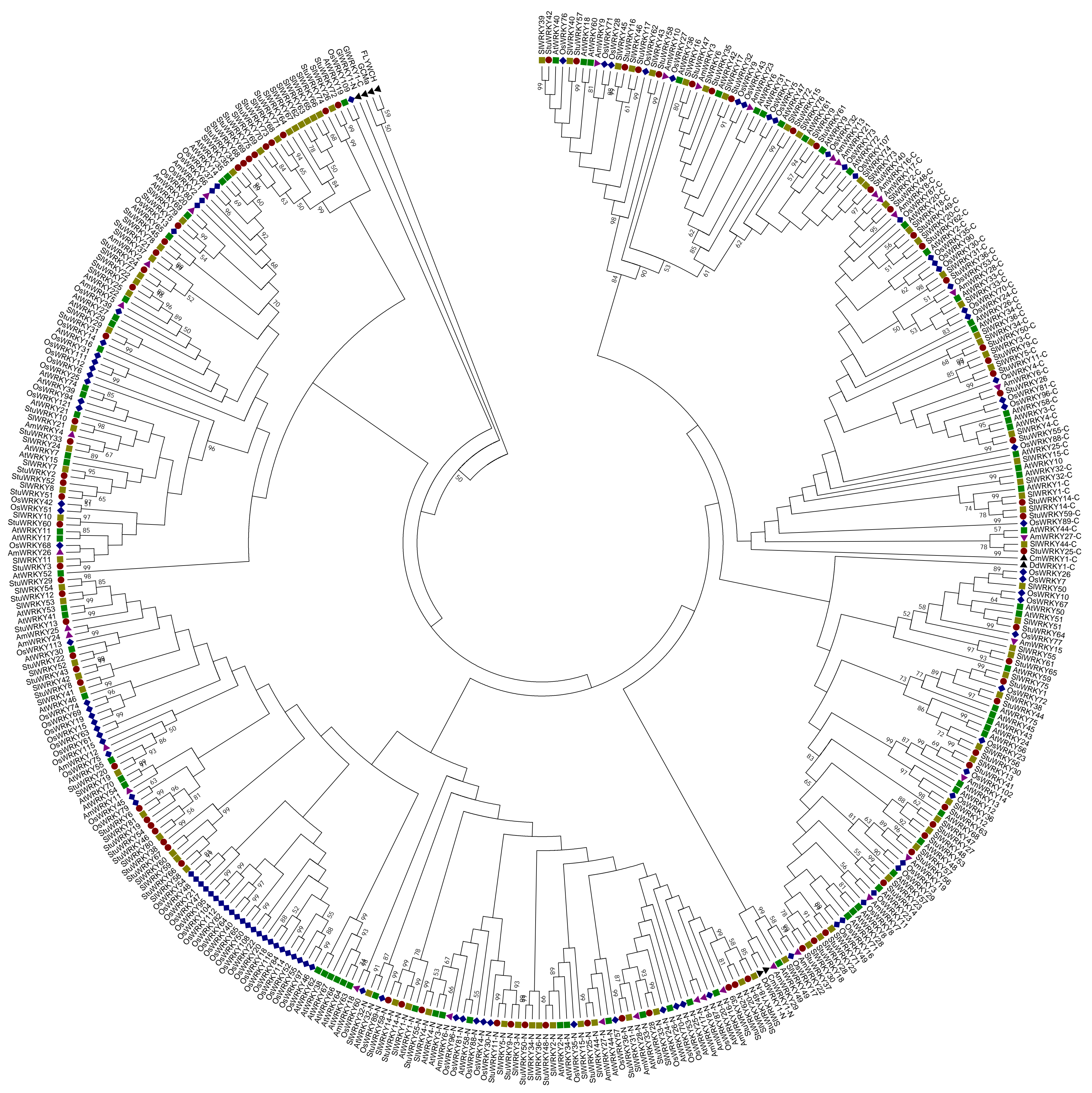

Supplement: Supplementary file 9 — Additional file 9: Figure S4: The phylogenetic tree with A. thaliana (green square), A. trichopoda (purple triangle), O. sativa (blue diamond), S. lycopersicum (gold square), and S. tuberosum (red dot) was constructed in MEGA5 using the Neighbor-Joining method with P-distance substitution model and a bootstrap value of 2000. Proteins used as an outgroup are indicated by a black triangle. WRKY domain alignment was performed with ClustalW. (PDF 691 KB) [file 12864_2013_6239_MOESM9_ESM.pdf]

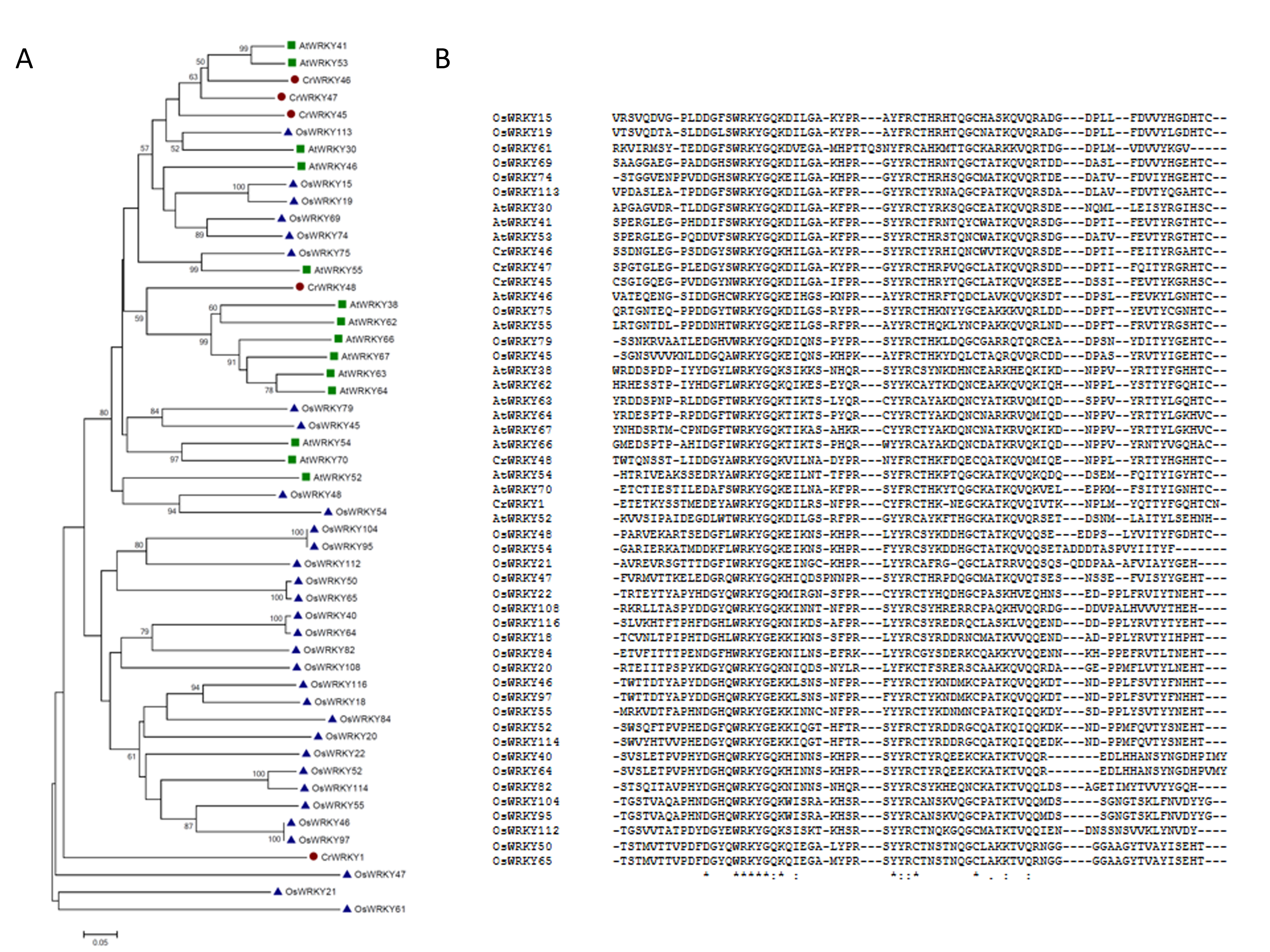

Supplement: Supplementary file 11 — Additional file 11: Figure S5: A-B. A. The phylogenetic relationship and alignment of CrWRKY1 to other group III WRKY TFs. The phylogenetic tree was constructed in MEGA5 using the Neighbor-Joining method with P-distance substitution model and a bootstrap value of 2000. WRKY domain alignment was performed with ClustalW. B. Alignment of the closest related rice WRKY genes and Catharanthus group III WRKYs was performed using ClustalW. (TIFF 11 MB) [file 12864_2013_6239_MOESM11_ESM.tiff]
