# Supplementary material for: Analyses of Catharanthus roseus and Arabidopsis thaliana WRKY transcription factors reveal involvement in jasmonate signaling
Source: BMC Genomics. 2014 Jun 20;15(1):502. doi: 10.1186/1471-2164-15-502 (PMC4099484; doi:10.1186/1471-2164-15-502)
Supplement: Supplementary file 6 — Additional file 6: Table S5: WRKY domain containing proteins were identified using 4 sources: manual searching, PlantTFcat, NCBI CDD, and MPGR. Rows indicate overlap in genes identified from the different sources. The bottom row provides a total number of genes identified by each method. (DOCX 15 KB) [file 12864_2013_6239_MOESM6_ESM.docx]

**Supplemental Table 5. WRKY domain containing proteins were identified using 4 sources:**

**manual searching, PlantTFcat, NCBI CDD, and MPGR.**

| **Gene** | **Manual WRKY Search** | **PlantTFcat** |  | **NCBI CDD** | **MPGR** |
| --- | --- | --- | --- | --- | --- |
| Cra549 | X | X |  | X |  |
| Cra1311 | X | X |  | X | X |
| Cra1702 | X | X |  | X | X |
| Cra2068 | X | X |  | X | X |
| Cra2271 | X | X |  | X |  |
| Cra2950 | X | X |  | X | X |
| Cra3503 | X | X |  | X | X |
| Cra3760 | X | X |  | X | X |
| Cra3799 | X | X |  | X | X |
| Cra4234 | X | X |  | X | X |
| Cra5093 | X | X |  | X | X |
| Cra5497 | X | X |  | X | X |
| Cra6088 | X | X |  | X | X |
| Cra6519 | X | X |  | X | X |
| Cra7867 | X | X |  | X | X |
| Cra8145 | X | X |  | X | X |
| Cra8670 | X | X |  | X | X |
| Cra9152 | X | X |  | X | X |
| Cra9369 | X | X |  | X | X |
| Cra10348 | X | X |  | X | X |
| Cra11684 | X | X |  | X |  |
| Cra13263 | X | X |  | X |  |
| Cra13321 | X | X |  | X | X |
| Cra16284 | X | X |  | X |  |
| Cra16307 | X | X |  | X |  |
| Cra17347 | X | X |  | X |  |
| Cra18915 | X | X |  | X | X |
| Cra18989 | X | X |  | X | X |
| Cra19330 | X | X |  | X | X |
| Cra19395 | X | X |  | X | X |
| Cra19580 | X | X |  | X | X |
| Cra20290 | X | X |  | X | X |
| Cra21821 | X | X |  | X | X |
| Cra22395 | X | X |  | X |  |
| Cra22725 | X | X |  | X | X |
| Cra23742 | X | X |  | X |  |
| Cra24943 | X | X |  | X | X |
| Cra28262 | X | X |  | X | X |
| Cra30069 | X | X |  | X | X |
| Cra37309 | X | X |  | X |  |
| Cra43671 | X | X |  | X |  |
| Cra43896 | X | X |  | X | X |
| Cra56567 | X | X |  | X | X |
| Cra65443 | X | X |  | X | X |
| Cra70197 | X | X |  | X | X |
| Cra105225 | X | X |  | X | X |
| Cra22691 |  | X |  | X | X |
| Cra24719 |  | X |  | X | X |
| Cra54213 |  | X |  | X | X |
| Cra55720 |  | X |  | X | X |
| Cra102390 |  | X |  | X | X |
| Cra11128 |  |  |  | X |  |
| Cra5637 |  |  |  |  | X |
| Cra10341 |  |  |  |  | X |
| Cra15757 |  | X |  |  |  |
| Cra16285 |  |  |  |  | X |
| Cra53604 |  |  |  |  | X |
| Cra72531 |  |  |  |  | X |
| Cra76953 |  |  |  |  | X |
| Cra82407 |  |  |  |  | X |
| **Total** | 46 | 52 |  | 52 | 47 |

Rows indicate overlap in genes identified from the different sources. The bottom row provides a total number of genes identified by each method.
