# Supplementary material for: Analyses of Catharanthus roseus and Arabidopsis thaliana WRKY transcription factors reveal involvement in jasmonate signaling
Source: BMC Genomics. 2014 Jun 20;15(1):502. doi: 10.1186/1471-2164-15-502 (PMC4099484; doi:10.1186/1471-2164-15-502)
Supplement: Supplementary file 10 — Additional file 10: Table S6: Catharanthus contained at least 48 WRKY TFs with 56 WRKY domains. The 70 amino acid sequence of each WRKY domain is provided. (DOCX 22 KB) [file 12864_2013_6239_MOESM10_ESM.docx]

**Supplemental Table 10. *Catharanthus* contained at least 48 WRKY TFs with 56 WRKY domains.**

| **WRKY Name** | **WRKY Domain** |
| --- | --- |
| CrWRKY1 | ETETKYSSTMEDEYAWRKYGQKDILRSNFPRCYFRCTHKNEGCKATKQVQIVTKNPLMYQTTYFGQHTCN |
| CrWRKY10-C | VHAAGDVGISGDGYRWRKYGQKMVKGNPHPRNYYRCTSAGCTVRKHIEMAKDNSNGVIITYKGRHDHDMP |
| CrWRKY10-N | FSVPAQKTPYPDGYNWRKYGQKQVKSPQGSRSYYRCTYSKCSAKKIECSDNSNRVIEIVYRSCHNHDPPE |
| CrWRKY11 | LQIESEIDVLDDGYRWRKYGQKVVKGNPNPRSYYKCTSAGCPVRKHVERASEDIKSVITTYEGKHNHEVP |
| CrWRKY12-C | VQTMSEVDVINDCYRWRKYGQKLVKGNPNPRSYYRCSNSGCPVKKHVERSSHDPKIVITTYEGKHDHEMP |
| CrWRKY12-N | VSPRIQEKALDDGYNWRKYGQKLVKGNVFVRSYYKCTYASCTSKKQVERSYDGRLTDIKYIGKHEHPKPQ |
| CrWRKY13 | TEASDTSLIVKDGYQWRKYGQKVTRDNPSPRAYFKCSFAPSCPVKKKVQRSIEDQSILVATYEGEHNHPH |
| CrWRKY14 | TDPDDKSLVVKDGYHWRKYGQKVTKDNPSPRAYFKCSFAPTCQVKKKVQRSVGNAAILVATYEGEHNHQP |
| CrWRKY15 | TEASDTSLIVKDGYQWRKYGQKVTRDNPSPRAYFKCSFAPSCPVKKKVQRSIEDQSIVVATYEGEHNHSK |
| CrWRKY16 | VRARSEAPMISDGCQWRKYGQKMAKGNPCPRAYYRCTMGVGCPVRKQVQRCAEDRSILITTYEGHHNHPL |
| CrWRKY17 | VRARCETATMNDGCQWRKYGQKIAKGNPCPRAYYRCTVAPTCPVRKQVQRCAEDTSILITTYEGTHNHSL |
| CrWRKY18 | VRVRCDTPTMNDGCQWRKYGQKIAKGNPCPRAYYRCTVAPNCPVRKQVQRCAEDMSILITTYEGTHNHTL |
| CrWRKY19 | VRARCETATMNDGCQWRKYGQKIAKGNPCPRAYYRCTVAPGCPVRKQVQRCLEDMSILITTYEGTHNHPL |
| CrWRKY20 | VRARSEAPMITDGCQWRKYGQKMAKGNPCPRAYYRCTMAAGCPVRKQVQRCADDRTILITTYEGNHNHPL |
| CrWRKY21 | FQTRSDVDVLDDGYKWRKYGQKVVKNSLHPRSYYRCTHNNCRVKKRVERLSEDCRMVITTYEGRHNHTPC |
| CrWRKY22 | FTTKSEIDHLEDGYRWRKYGQKAVKNSPFPRSYYRCTSQKCSVKKRVERSFQDPSIVITTYEGQHNHHCP |
| CrWRKY23 | FRTKSQVEILDDGYKWRKYGKKMVKNSPNPRITTDAQLKDAPVKKRVERDKEDPKYVITAYEGIHNHQGP |
| CrWRKY24 | FKTLSDVDVLDDGYKWRKYGQKVVKNTQHPRSYYRCTQDNCRVKKRVERLAEDPRMVITTYEGRHIHSPS |
| CrWRKY25 | RIKSCDSAMTDDGYKWRKYGQKSIKNSPNPRSYYRCTNPRCAAKKQVERSSDDPDTLIITYEGLHLHFAY |
| CrWRKY26 | FMTKSDVDHLEDGYRWRKYGQKAVKNSPFPRSYYRCTSASCNVKKRVERCLNDPSLVITTYEGQHNHQTP |
| CrWRKY27 | FMTKSEVDHLEDGYRWRKYGQKAVKNSPFPRSYYRCTNTKCTVKKRVERSSEDPTIVITTYEGQHCHHTV |
| CrWRKY28 | FQTRSQVDILDDGYRWRKYGQKAVKNNKFPRSYYRCTYQGCNVKKQVQRLSKDEGIVVTTYEGMHSHPIE |
| CrWRKY29 | FMTKSEVDHLEDGYRWRKYGQKAVKNSPYPRSYYRCTTQKCPVKKRVERSFQDPSIVITTYEGTHNHHVP |
| CrWRKY2-C | VQTTSEVDLLDDGYRWRKYGQKVVKGNPYPRSYYKCTSPGCNVRKHVERAATDPKAVITTYEGKHNHDVP |
| CrWRKY2-N | QASILVDKPADDGYNWRKYGQKQVKGSEYPRSYYKCTHQNCPVKKKVERSQDGQVTEIIYKGQHNHPPPQ |
| CrWRKY30 | FHTRSTEDILDDGYKWRKYGQKSVKNSSHPRSYYRCTHHTCNVKKQIQRLSKDTSVVVTTYEGIHSHPCE |
| CrWRKY31 | FQTRSADDVLDDGYRWRKYGQKSVKNSKYPRSYYRCSQHTCNVKKQVQRLSKDTGIVVTTYEGIHNHPCE |
| CrWRKY32 | FKTKSDVEILDDGFKWRKYGRKMVKNSINPRNYYKCSVEGCPVKKRVERDNNDSRYVVTTYEGIHNHQGP |
| CrWRKY33 | FMTKSEIDQLDDGFRWRKYGQKAVKNSPFPRSYYRCTTAGCGVKKRVERSSEDATIVITTYEGMHNHCSP |
| CrWRKY34 | AISMKMADIPPDDYSWRKYGQKPIKGSPHPRGYYKCSSVRGCPARKHVERALDDPSMLIVTYEGEHNHSL |
| CrWRKY35 | AISMKMADIPPDDYSWRKYGQKPIKGSPHPRGYYKCSSVRGCPARKHVERALDDPTMLIVTYEGEHNHSH |
| CrWRKY36 | AISNKLADIPPDEYSWRKYGQKPIKGSPHPRGYYKCSSMRGCPARKHVERCLEDPSMLIVTYEGEHNHPR |
| CrWRKY37 | AISSKIADIPADEYSWRKYGQKPIKGSPYPRGYYKCSTVRGCPARKHVERATDDPKMLIVTYEGEHRHVQ |
| CrWRKY38 | SRNRTEVYPPPDSWSWRKYGQKPIKGSPYPRGYYRCSSSKGCPARKQVERSRLDPTKLLITYSSEHNHSL |
| CrWRKY39 | RLKGEMGAPPSDSWAWRKYGQKPIKGSPYPRGYYRCSSSKGCPARKQVERSRIDPTMLMVTYTCEHNHPW |
| CrWRKY3-C | VQTVSEVDILDDGYRWRKYGQKVVRGNPNPRSYYKCTNAGCPVRKHVERASHDPKAVITTYEGKHNHNVP |
| CrWRKY3-N | TSSITSDRSSDDGYNWRKYGQKLVKGSEFPRSYYKCTYPNCEVKKIFERSPDGQITEIVYKGSHDHPKPQ |
| CrWRKY40 | DKKQKKEGPPLDCWSWRKYGQKPIKGSPYPRGYYRCSTSKGCSAKKQVERCRTDPTVLIVTYTSTHNHAT |
| CrWRKY41 | VCQVPAEALSSDTWSWRKYGQKPIKGSPYPRGYYRCSTSKGCLARKQVERNRSDPGMFIVTYTAEHNHPM |
| CrWRKY42 | SRPSSGEVVPSDLWAWRKYGQKPIKGSPYPRGYYRCSSSKGCSARKQVERSRNDPNMLVITYTSEHNHPW |
| CrWRKY43 | VHQMTQEELSGDSWAWRKYGQKPIKGSPYPRNYYRCSTSKGCSARKQVERCPTDPNIFVVSYSGEHTHPR |
| CrWRKY44 | VIQVTAEDLSSDKWAWRKYGQKPIKGSPYPRSYYRCSSSKGCLARKQVEQSCKDPSIFIVTYTAEHSHSQ |
| CrWRKY45 | CSGIGQEGPVDDGYNWRKYGQKDILGAIFPRSYYRCTHRYTQGCLATKQVQKSEEDSSIFEVTYKGRHSC |
| CrWRKY46 | SSDNGLEGPSDDGYSWRKYGQKHILGAKYPRSYYRCTYRHIQNCWVTKQVQRSDEDPTIFEITYRGAHTC |
| CrWRKY47 | SPGTGLEGPLEDGYSWRKYGQKDILGAKYPRGYYRCTHRPVQGCLATKQVQRSDDDPTIFQITYRGRHTC |
| CrWRKY48 | TWTQNSSTLIDDGYAWRKYGQKVILNADYPRNYFRCTHKFDQECQATKQVQMIQENPPLYRTTYHGHHTC |
| CrWRKY4-C | VQTRSEVDLLDDGYKWRKYGQKVVKGNPHPRSYYRCTYAGCNVRKHVERASTDAKAVVTTYEGKHNHDIP |
| CrWRKY4-N | VAAVALDKPADDGYNWRKYGQKLVKAKEHPRSYYKCTHLNCPVKKKVERATDGHVAEITYKGQHNHEMPQ |
| CrWRKY5-C | VQTTSDIDILDDGYRWRKYGQKVVKGNPNPRSYYKCTYAGCPVRKHVERASHDLRAVITTYEGKHNHDVP |
| CrWRKY5-N | SQYLREQRKSEDGYNWRKYGQKQVKGSENPRSYYKCTFPSCPTKKKVERNLEGHITEIVYKGNHNHAKPQ |
| CrWRKY6-C | VQNTVDSEIIRDGFRWRKYGQKVVKGNPYPRSYYRCTSLKCNVRKYVERTSEDPTAFITTYEGKHNHEMP |
| CrWRKY6-N | SHSTLGDRPSYDGYNWRKYGQKQVKGSEYPRSYYKCTHPNCPVKKKVERSLDGQIAEIVYKGEHNHPKPQ |
| CrWRKY7-C | VQTTSEVDILDDGYRWRKYGQKVVKGNPNPRSYYKCTSAGCTVRKHVERASHDLKSVITTYEGKHNHDVP |
| CrWRKY7-N | GDPNIGGAPAEDGYNWRKYGQKQVKGSEYPRSYYKCTHQNCQVKKKVERSQEGHITEIIYKGAHNHPKPP |
| CrWRKY8-C | VQTTSDIDILDDGYRWRKYGQKVVKGNPNPRSYYKCTSPGCPVRKHVERASHDLRSVITTYEGKHNHDVP |
| CrWRKY8-N | QQTMSERRRAEDGYNWRKYGQKNVKGSENPRSYYKCTFPSCPTKKKVERSVDGQITEIVYKGNHNHAKPQ |
| CrWRKY9-C | VQTMSEVDVINDCYRWRKYGQKLVKGNPNPRSYYRCSNSGCPVKKHVERSSHDPKIVITTYEGKHDHEMP |
| CrWRKY9-N | VSPRIQEKALDDGYNWRKYGQKLVKGNVFVRSYYKCTYASCTSKKQVERSYDGRLTDIKYIGKHEHPKPQ |
