# Supplementary material for: Analyses of Catharanthus roseus and Arabidopsis thaliana WRKY transcription factors reveal involvement in jasmonate signaling
Source: BMC Genomics. 2014 Jun 20;15(1):502. doi: 10.1186/1471-2164-15-502 (PMC4099484; doi:10.1186/1471-2164-15-502)

**Supplemental Table 7. A list of primers used in qRT-PCR to measure transcript levels.**

| **qRT-PCR Primer** | **Gene** | **Primer Sequence** |
| --- | --- | --- |
| Cr3894-LP | EF1a | TACTGTCCCTGTTGGTCGTG |
| Cr3894-RP | EF1a | AAGAGCTTCGTGGTGCATCT |
| JAZ2-FW | JAZ2 | ACGGAGGTGAAGTTTTGGTG |
| JAZ2-RV | JAZ2 | CATCGGGAGAATGGTGTTTC |
| G10H-FW | G10H | TTATTCGGATTCTGCCAAGG |
| G10H-RV | G10H | TCCCCAAAGTGAATCGTCAT |
| TDC-FW | TDC | ATCCGATCAAACCCATACCA |
| TDC-RV | TDC | CGTCATCCTCGACCATTTTT |
| STR-FW | STR | ACCATTGTGTGGGAGGACAT |
| STR-RV | STR | ATTTGAATGGCACTCCTTGC |
| Cr4234-FP1 RT | CrWRKY3 | ATCACATGATCATCCAAAACCTC |
| Cr4234-RP1 RT | CrWRKY3 | GCCAGTAAAAGACGAATCCCTAT |
| Cr6088-FP1 RT | CrWRKY5 | ATTTCCACCTTCTTCTCTTCCAA |
| Cr6088-RP1 RT | CrWRKY5 | AGAACAGGGGAATCAAGAAGAAC |
| Cr10348-FP1 RT | CrWRKY8 | TTAGCTGATGATGATTTTGAGCA |
| Cr10348-RP1 RT | CrWRKY8 | CCTTCACTTTCAAACTCCCTTTT |
| Cr22691-FP1 RT | CrWRKY11 | TCCTCAAATCCAGAAATACAAGTTC |
| Cr22691-RP1 RT | CrWRKY11 | CTTCCACTGGAGTTCATAACAAAAT |
| Cr1311-FP1 RT | CrWRKY13 | GGAAGAAAAATGCCTCTTGTTAAA |
| Cr1311-RP1 RT | CrWRKY13 | ATCAAATGTTTTCTCAAAGCATCA |
| Cr3503-FP1 RT | CrWRKY17 | CTTCTTCAAATTCTCAGTCTGCAA |
| Cr3503-RP1 RT | CrWRKY17 | TCTAAAGTAATGGTGGGAAATGGT |
| Cr18915-FP1 RT | CrWRKY18 | TCCATGTTAATCTCTGGTTCTTCA |
| Cr18915-RP1 RT | CrWRKY18 | AATGGATGAATTTGGAAGGTAAAA |
| Cr2271-FP1 RT | CrWRKY21 | TTCTTAGCTCCTTCTTCTTCACAAA |
| Cr2271-RP1 RT | CrWRKY21 | CAAGAGTTCATGACTATGGTTGTTG |
| Cr19330-FP1 RT | CrWRKY26 | TCTGATCAGTAGTAACCCCTCCTC |
| Cr19330-RP1 RT | CrWRKY26 | ACTGCTTGATAAGGAACCAAAGTC |
| Cr24943-FP2 RT | CrWRKY28 | GGATCAAATGGGCTGTTAG |
| Cr24943-RP2 RT | CrWRKY28 | TCGGAGCTGATGATGATTC |
| Cr3760-FP1 RT | CrWRKY35 | GCCGGTAAATTCTTTTATGTCATC |
| Cr3760-RP1 RT | CrWRKY35 | AGACGTGTGAGGACAGATTTGTAA |
| Cr7867-FP1 RT | CrWRKY36 | GAAATGATGTATAGACGCAGCAAC |
| Cr7867-RP1 RT | CrWRKY36 | CACTCCCATCAATACTCAATGAAG |
| Cr11684-FP1 RT | CrWRKY38 | ATCAGAAGAATTTGGAGTTTTTGC |
| Cr11684-RP1 RT | CrWRKY38 | AACACAATAGAAGATGATCCCACA |
| Cr20290-FP1 RT | CrWRKY41 | AAGTTGGAATTAGAGAGCAGCAGT |
| Cr20290-RP1 RT | CrWRKY41 | GATGATTTTTCAGCCAATTCTTCT |
| Cr3799-FP1 RT | CrWRKY45 | TGTTAATGAGCTGCTATTCTCCAG |
| Cr3799-RP1 RT | CrWRKY45 | TCATTTGTCACTGAATTTGGAGTT |
| Cr24719-FP1 RT | CrWRKY48 | TTCCCAAACCAATTTTCTGATTAT |
| Cr24719-RP1 RT | CrWRKY48 | AACGTATGAGTACTTGCCGTACAA |
| Cr55720-FP1 RT | CrWRKY49 | ATATAAGAGACAAAATCGTCGATGC |
| Cr55720-RP1 RT | CrWRKY49 | ATTTTCCAGCCTGATTACTCTGTT |
| Cr56567-FP1 RT | CrWRKY50 | CTAGTCTCAACAACAAGAACAACAAGA |
| Cr56567-RP1 RT | CrWRKY50 | ATTGATAACCATCCTTCACAATAAGAG |
| Cr65443-FP1 RT | CrWRKY51 | ACAGTAGCTGAATCCAAGATTGTTC |
| Cr65443-RP1 RT | CrWRKY51 | TTTATTCCTCGTTCCTCTATTCCTAA |
| Cr70197-FP1 RT | CrWRKY52 | CACAAGTATGACCAAGGATGTGTAG |
| Cr70197-RP1 RT | CrWRKY52 | GTAGTGATGATTCGCTCAACTTTTT |


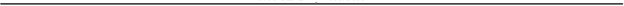

Supplement: Supplementary file 12 — Additional file 12: Table S7: A list of primers used in qRT-PCR to measure transcript levels. (DOCX 14 KB) [file 12864_2013_6239_MOESM12_ESM.docx]
