# Supplementary material for: Analyses of Catharanthus roseus and Arabidopsis thaliana WRKY transcription factors reveal involvement in jasmonate signaling
Source: BMC Genomics. 2014 Jun 20;15(1):502. doi: 10.1186/1471-2164-15-502 (PMC4099484; doi:10.1186/1471-2164-15-502)

**Supplemental Table 8. A list or primers used in cloning to isolate full WRKY domains for those TFs in the *Catharanthus* MPGR database with partial domain sequences.**

| **Primer** | **Gene** | **Primer Sequence** | **Notes** |
| --- | --- | --- | --- |
| T7 |  | TAATACGACTCACTATAGGG | pGEM-T Vector LP |
| SP6 |  | ATTTAGGTGACACTATAGAAT | pGEM-T Vector RP |
| 5' AAP |  | GGCCACGCGTCGACTAGTACGGGIIGGGIIGGGIIG | 5' RACE Adapter/1st Primer |
| 5' AUAP |  | GGCCACGCGTCGACTAGTAC | 5' RACE 2nd Primer |
| 3' AP |  | GGCCACGCGTCGACTAGTTTTTTTTTTTTTTTTT | 3' RACE Adapter/1st Primer |
| 3' AUAP |  | GGCCACGCGTCGACTAGTAC | 3' RACE PCR Primer/2nd Primer |
| Cr10348-LP-Insert | CrWRKY8 | CTTATCAGAGCTTTTCCCCTGA | Verify presence of insertion |
| Cr10348-RP-Insert | CrWRKY8 | ACCACTCTTTTGAGAGCTTTGC | Verify presence of insertion |
| Cr11128-LP1-3’ | CrWRKY9 | CCATGAAAATGGATACCATGC | 3' RACE to sequence Cr11128, 1st Primer |
| Cr11128-LP2-3’ | CrWRKY9 | AATGCCAAAGCAAGAGTCAGA | 3' RACE to sequence Cr11128, 2nd Primer |
| Cr11128-LP3-3' | CrWRKY9 | TTGTACCAAAACCAGATAAAGATGG | 3' RACE to sequence Cr11128, 3rd Primer |
| Cr11128-LP4-3' | CrWRKY9 | TGTAGATTTCCAAAGTACCAACACC | 3' RACE to sequence Cr11128, 4th Primer |
| Cr11128-LP5-3' | CrWRKY9 | ACATATGCTAGTTGCACATCAAAAA | 3' RACE to sequence Cr11128, 5th Primer |
| Cr22691-RP1-5’ | CrWRKY11 | GCGAAAAATCATTTGGTTTCA | 5' RACE to sequence Cr22691, 1st Primer |
| Cr22691-RP2-5’ | CrWRKY11 | AGGCAATGTCAGGATTGAATG | 5' RACE to sequence Cr22691, 2nd Primer |
| Cr22691-RP2.5-5' | CrWRKY11 | GGGTAGACCTTGAAGGCAAATA | 5' RACE to sequence Cr22691, 2.5 Primer |
| Cr22691-RP3-5’ | CrWRKY11 | CAGCTGAGAAGATTTCTTTCTTCC | 5' RACE to sequence Cr22691, 3rd Primer |
| Cr22691-RP4-5’ | CrWRKY11 | GATTTCTTTCTTCCTCCTTTTTGG | 5' RACE to sequence Cr22691, 4th Primer |
| Cr43671-RP1-5' | CrWRKY12 | TTCTTGAGGTGGAACAAATGC | 5' RACE to sequence Cr43671, 1st Primer |
| Cr43671-RP2-5' | CrWRKY12 | AAATGCCGAAATAGGTGGACT | 5' RACE to sequence Cr43671, 2nd Primer |
| Cr54213-RP1-5’ | CrWRKY15 | GCAAGTGCTGCTTTGAAACTT | 5' RACE to sequence Cr54213, 1st Primer |
| Cr54213-RP2-5’ | CrWRKY15 | GCCACCACAATTGATTGATCT | 5' RACE to sequence Cr54213, 2nd Primer |
| Cr24719-RP1-5' | CrWRKY48 | ATCCAACCACCATATGATCCA | 5' RACE to sequence Cr24719, 1st Primer |
| Cr24719-RP2-5' | CrWRKY48 | AAACGCTGTCGTTTCGTTAGA | 5' RACE to sequence Cr24719, 2nd Primer |
| Cr55720-RP1-5’ | CrWRKY49 | GAAGCCATCTGTTCCACAAAG | 5' RACE to sequence Cr55720, 1st Primer |
| Cr55720-RP2-5’ | CrWRKY49 | TAATTGTCGGACCAGATGAGC | 5' RACE to sequence Cr55720, 2nd Primer |
| Cr56567-LP1-3' | CrWRKY50 | AGCCAAATGGTGTAGCAGATG | 3' RACE to sequence Cr56567, 1st Primer |
| Cr56567-LP2-3' | CrWRKY50 | GCCAAGGTTTCAATTGTTTCA | 3' RACE to sequence Cr56567, 2nd Primer |
| Cr65443-RP1-5' | CrWRKY51 | CGTCTTCGTTCTCTTTGCATC | 5' RACE to sequence Cr65443, 1st Primer |
| Cr65443-RP2-5' | CrWRKY51 | ATCGTGCCCCTCATCTATTTC | 5' RACE to sequence Cr65443, 2nd Primer |
| Cr70197-RP1-5’ | CrWRKY52 | GATTCGCTCAACTTTTTGCAG | 5' RACE to sequence Cr70197, 1st Primer |
| Cr70197-RP2-5’ | CrWRKY52 | CGTCTTGGACCTTTTGGATTT | 5' RACE to sequence Cr70197, 2nd Primer |


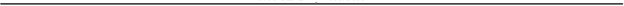

Supplement: Supplementary file 13 — Additional file 13: Table S8: A list or primers used in cloning to isolate full WRKY domains for those TFs in the Catharanthus MPGR database with partial domain sequences. (DOCX 15 KB) [file 12864_2013_6239_MOESM13_ESM.docx]
